# Antioxidant Potential of Propolis, Bee Pollen, and Royal Jelly: Possible Medical Application

**DOI:** 10.1155/2018/7074209

**Published:** 2018-05-02

**Authors:** Joanna Kocot, Małgorzata Kiełczykowska, Dorota Luchowska-Kocot, Jacek Kurzepa, Irena Musik

**Affiliations:** Department of Medical Chemistry, Medical University of Lublin, 4A Chodźki Street, 20-093 Lublin, Poland

## Abstract

Honeybees products comprise of numerous substances, including propolis, bee pollen, and royal jelly, which have long been known for their medicinal and health-promoting properties. Their wide biological effects have been known and used since antiquity. Bee products are considered to be a potential source of natural antioxidants such as flavonoids, phenolic acids, or terpenoids. Nowadays, the still growing concern in natural substances capable of counteracting the effects of oxidative stress underlying the pathogenesis of numerous diseases, such as neurodegenerative disorders, cancer, diabetes, and atherosclerosis, as well as negative effects of different harmful factors and drugs, is being observed. Having regarded the importance of acquiring drugs from natural sources, this review is aimed at updating the current state of knowledge of antioxidant capacity of selected bee products, namely, propolis, bee pollen, and royal jelly, and of their potential antioxidant-related therapeutic applications. Moreover, the particular attention has been attributed to the understanding of the mechanisms underlying antioxidant properties of bee products. The influence of bee species, plant origin, geographic location, and seasonality as well as type of extraction solutions on the composition of bee products extracts were also discussed.

## 1. Introduction

Bee products like propolis, bee wax, pollen, royal jelly, as well as honey had been known and used even in antiquity and the Middle Ages. For example, in ancient China, bee pollen was applied as a cosmetic agent contributing to skin whitening. At present, these substances are applied in a branch of complementary and alternative medicine—apitherapy. Moreover, the interest in their use as agents in the cure of cancers, neurodegenerative, cardiovascular, and gastrointestinal tract diseases as well as the treatment of wounds and burns has still been growing [1–10].

Bee products are considered to be a potential source of natural antioxidants capable of counteracting the effects of oxidative stress underlying the pathogenesis of numerous diseases.

In general, the compounds possessing phenolic character, which belong to substances expressing ability to scavenge free radicals, are mainly responsible for bee products' antioxidant capacity [11–14]. They comprise of two main groups of compounds—flavonoids and phenolic acids [15].

**Figure 1:**
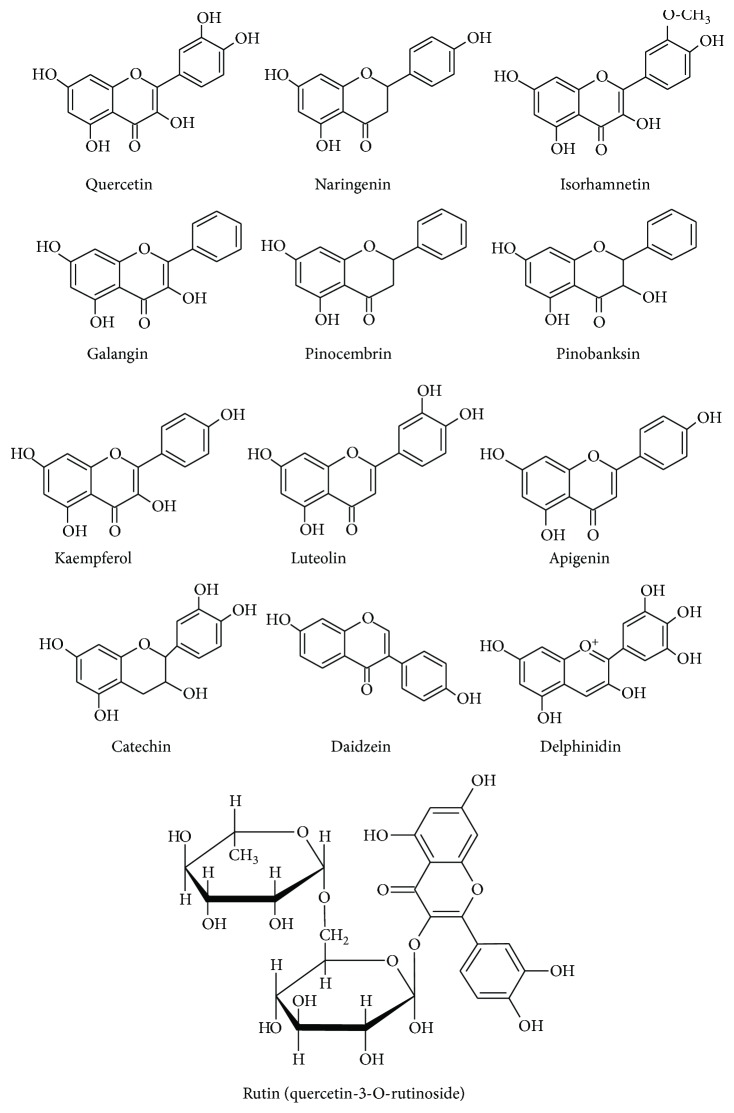
The examples of flavonoids and their glycosides detected in bee products. Quercetin, naringenin, isorhamnetin, and kaempferol: detected in propolis, bee pollen, royal jelly; galangin and pinocembrin: detected in propolis and bee pollen; pinobanksin: detected in propolis and royal jelly; luteolin, apigenin, and rutin: detected in propolis, bee pollen, and royal jelly; catechin and delphinidin: detected in bee pollen; daidzein: detected in propolis [29, 32, 74, 78, 83, 103].

Flavonoids are plant derivatives of polyphenolic structure comprising several subgroups like flavones, flavonols, flavanones flavanonols, flavanols (catechins), anthocyanins, and chalcones, as well as isoflavones and neoflavonoids. The best known subgroups are the compounds containing benzo-*γ*-pyrone skeleton. Flavonoids often occur in the form of glycosides, in which they play a role of aglycones connected by a glycosidic bond with a carbohydrate group [15–17]. The presence of phenol groups in the molecules of flavonoids imparts them the antiradical activity all the more because the radicals formed during scavenging are resonance stabilized [16]. The examples of flavonoids and their glycosides found in bee products are presented in Figure 1.

**Figure 2:**
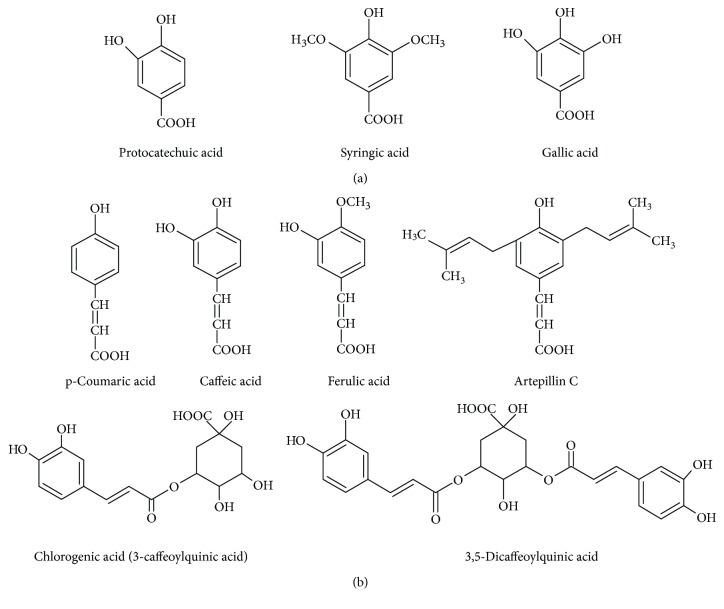
The examples of phenolic acids and their derivatives found in bee products: (a) benzoic acid derivatives and (b) cinnamic acid derivatives. Protocatechuic acid, syringic acid, gallic acid, *p*-coumaric acid: detected in propolis and bee pollen; caffeic acid and ferulic acid: detected in propolis, bee pollen, and royal jelly; artepillin C, chlorogenic acid, and 3,5-dicaffeoylquinic acid: detected in propolis [28, 41, 71, 74, 78, 102, 139].

The phenolic acids are compounds possessing carboxylic and phenol groups. Recently, a growing concern in their possible application for human health protection has been observed, considering their antioxidant activities including the prevention of oxidation processes and generation of oxygen species as well as chelating prooxidative metals [18]. The examples of phenolic acids and their derivatives found in bee products are presented in Figure 2.

**Figure 3:**
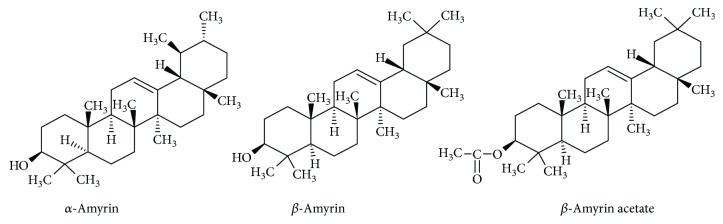
The examples of amyrins found in propolis [19, 20].

To compounds without phenolic character being responsible for the antioxidant capacity of propolis (especially Brazilian one) belong amyrins [19, 20]. *α*- and *β*-amyrins belong to triterpenoids of plant origin. They have been reported to exhibit numerous beneficial properties including antiapoptotic, antioxidant, anti-inflammatory, and antifibrotic as well as gastro- and hepatoprotective effects. Studies have revealed the possible application of *β*-amyrin in Parkinson's disease therapy [21, 22]. The examples of amyrins found in bee propolis are presented in Figure 3.

**Figure 4:**
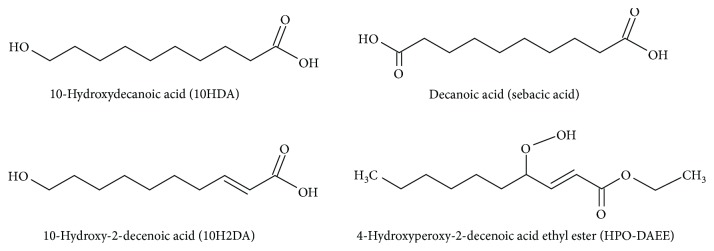
The main carboxylic acids of the royal jelly and their derivatives [23, 24, 105].

Considering the antioxidant activity of royal jelly hydroxy dicarboxylic fatty acids with 8–12 carbon atoms in the chain and their derivatives is worth mentioning. The major fatty acid is 10-hydroxydecanoic acid (10-HDA) whose presence has not been reported in any other natural raw material or even in any other product of apiculture [23]. Other carboxylic acids included in RJ are 10-hydroxy-2-decenoic acid (10H2DA) and sebacic acid (SA) [24] (Figure 4).

In scientific research, propolis and bee pollen extracts are used instead of the raw substance due to the fact that they contain higher amounts of bioactive components [2]. However, the application of solvents of different polarities affects the composition of the obtained extracts as the components of bee products possess diverse structures, and while hydrophilic ones are better soluble in polar solvents like alcohols, those with hydrophobic properties exhibit greater affinity to nonpolar solvents like hydrocarbons. The properties of the extract depend strongly not only on the solvent used but also on extraction conditions, that is, time and temperature as well [13, 25].

Having regarded the importance of acquiring drugs from natural sources, this review is aimed at updating the current state of the knowledge of antioxidant capacity of the selected bee products, namely, propolis, bee pollen, and royal jelly, and of their possible medical applications as natural substances capable of counteracting the effects of oxidative stress underlying pathogenesis of numerous diseases, such as neurodegenerative disorders, cardiovascular diseases, diabetes, and cancer, as well as negative effects of different harmful factors and drugs. Moreover, particular attention has been attributed to the understanding of the mechanisms underlying possible antioxidant properties of bee products. The influence of bee species, plant origin, geographic location, and seasonality, as well as type of extraction solutions on the composition of bee products extracts were all discussed.

## 2. Propolis: “Bee Glue”

Propolis, generally known as the “bee glue,” is a resinous mixture that honey bees produce by mixing their saliva containing certain enzymes and beeswax with exudate gathered mainly from leaf and flower buds, stems, and bark cracks of numerous species of trees. The word *propolis* is derived from two Greek words *pro* and *polis*, which mean “defense” and “city” or “community,” respectively. Bees use it mainly as a sealant and a disinfecting material. Propolis is used for sealing holes and cracks, smoothing the inner surface, and retaining internal temperature of the beehive as well as for preventing weathering (e.g., it is used for decreasing the size of the outlet opening during periods of cold weather) and invasion of predators [3, 26]. Due to its antimicrobial activity, it also contributes to an aseptic internal environment and is used to cover (“mummify”— to prevent decay) the body of dead pests that have invaded the hives (e.g., shrews and mice), which are too big to be removed outside [19].

### 2.1. Composition of Propolis

Raw propolis is typically composed of 50–60% of resins and balms (including phenolic compounds), 30–40% of waxes and fatty acids, 5–10% of essential oils, 5% of pollen, and about 5% of other substances including amino acids, micronutrients, and vitamins (thiamin, riboflavin, pyridoxine, C, and E) [20, 27]. According to the literature data, more than 300 compounds belonging to polyphenols, terpenoids, steroids, sugars, amino acids, and others have been identified in propolis [3, 28].

Propolis from the temperate zone all over the world (Europe, nontropic regions of Asia, North America, and continental Australia) is classified as the poplar type propolis since it originates mainly from the bud exudates of *Populus* spp., most often *P. nigra* L. The main biologically active components of this type of propolis are flavonoids (flavones and flavanones), phenolic acids (cinnamic acid), and their esters [27–29]. Birch propolis, found in Russia, originates from *Betula verrucosa* Ehrh. and similarly contains flavones and flavonols (but not the same as in poplar propolis) [29]. Mediterranean propolis is characteristic of subtropic regions like Greece, Greek islands, Sicily, Malta, Cyprus, Croatia, and Algeria. It originates mainly from the resin of *Cupressus sempervirens* (commonly known as the Mediterranean or Italian cypress) and is characterized by relatively high amounts of diterpenes [28, 29]. In tropical zones, in turn, several types of propolis derived from many different sources have been identified. For example, in Brazil, there are 13 different types of propolis including green, red, and brown ones, whose main sources are *Baccharis dracunculifolia*, *Dalbergia ecastaphyllum* [30], and *Hyptis divaricata*, respectively. The most popular is the green one, which owes its color to the chlorophyll occurring in young tissues and nonexpanded leaves of *B. dracunculifolia* and collected by the bees [31]. This type of propolis is rich in derivatives of phenylpropanoids (e.g., artepillin C) and diterpenes, whereas flavonoids occur in small amounts [29]. The red propolis is characterized by the presence of numerous flavonoids (formononetin, liquiritigenin, pinobanksin-3-acetate, pinobanksin, luteolin, rutin, quercetin, pinocembrin, daidzein, and isoliquiritigenin), which are found in the resinous exudates from the surface of *D. ecastaphyllum* [30, 32]. This type of propolis is also characteristic of Cuba and Mexico [33]. The brown propolis is mainly produced in northeastern Brazil from *H. divaricata* [31]. Other examples of tropical propolis include the one originating from resin exuded by the flowers of *Clusia* sp. found in Cuba and Venezuela—with its main constituents being derivatives of benzophenones—and “Pacific” propolis originating from the tropical tree *Macaranga tanarius* found on Pacific Ocean tropical islands (Taiwan, Okinawa, and Indonesia), which chemical makers are C-prenylflavanones [29, 33].

### 2.2. Relationships between the Antioxidant Capacity of Propolis and Its Composition

Antioxidant properties of propolis have been fully investigated and proven with the use of DPPH, ABTS^+^, FRAP, and ORAC methods [3, 19, 20, 29, 31, 34–38]. In the same *in vitro* studies, the antioxidant capacity of propolis extracts was found to be similar to that of the synthetic antioxidant butylated hydroxytoluene or ascorbic acid [4, 20]. Importantly, the antioxidant capacity of propolis is dependent on its content, but the studies aiming at finding the distinct relationships between these two parameters are not consistent [3, 35, 36]. In general, according to the literature data, the total phenolic content of propolis extracts ranged from about 30 to 200 mg of gallic acid equivalents (GAE)/g of dry weight, and the flavonoid content ranged from about 30 to 70 mg of quercetin equivalents (QE)/g, whereas DPPH free radical-scavenging activity ranged from about 20 to 190 *μ*g/mL [3, 19, 20, 31, 35, 36, 38]. The phenolic compounds, but the different ones than the flavonoids, are believed to be responsible for the antioxidant activity of Brazilian propolis. According to Zhang et al. [36], 3,4,5-tricaffeoylquinic acid, 3,5-dicaffeoylquinic acid, 4,5-dicaffeoylquinic acid, and artepillin C seem to be responsible for the strong antioxidant activity of Brazilian green propolis. Unlike in the case of Brazilian propolis, the antioxidant activity of poplar propolis appears to be largely influenced by both total polyphenol and total flavonoid contents [3, 34, 38]. The results obtained by Fabris et al. [39] indicated that European (Italy and Russia) propolis samples had similar polyphenolic composition and consequently similar antioxidant activity, while Brazilian propolis possessed lower polyphenolic amount and thus antioxidant properties. In general, there seems to be a big problem with the standardization of propolis composition—this results from the fact that this is highly dependent on many factors, such as bee species, plant origin, geographic location, temperature variation, and seasonality, as well as storage conditions [3, 19, 20, 27, 31, 36, 40]. Recently, for example, Bonamigo et al. [19, 20] have studied the antioxidant activity of the ethanol extract of Brazilian propolis samples (collected from the same region) depending on bees' species, *Scaptotrigona depilis*, *Melipona quadrifasciata anthidioides*, *Plebeiadroryana*, and *Apis mellifera.* The studied samples were shown to differ in composition as well as in both free radical-scavenging activity and ability to inhibit lipid peroxidation. In general, propolis obtained from *A. mellifera* revealed the highest activity. Calegari et al. [40], in turn, found that Brazilian propolis samples produced in both March and April showed a difference in color and higher content of total phenolic compounds as well as antioxidant capacity than those produced in May and June, which indicated that the chemical composition of propolis depended on the month of production—this effect can be explained by variations in temperature. The researchers also reported that bees' colonies, which received food supplementation every three days throughout the year, displayed significantly higher total phenolic and flavonoids content as well as antioxidant capacity than those bereaved of this supplementation [40].

Moreover, both the chemical composition and biological properties of propolis extracts are highly dependent on the type of solvents used for the extraction [3, 31, 34]. The most commonly used solvent for the extraction of propolis is aqueous ethanol (particularly at concentration of 70–75%), followed by others, such as ethyl ether, water, methanol, hexane, and chloroform. Sun et al. [3] showed that extraction yields (the ratio of the weight of the dry extract to the weight of the raw extract) of Beijing propolis ranged from 1.8% to 51% and exhibited the tendency to increase along with the enhancement of the ethanol concentration. The total polyphenol and total flavonoid content distinctly varied and ranged from 6.68 to 164.20 mg GAE/g and 4.07 to 282.83 mg of rutin equivalents (RE)/g, respectively, and the highest concentration was observed in 75% ethanol solvents, a little bit lower in 95% and 100% ethanol solvents and the lowest one in water solution. The 75% extract also demonstrated the highest antioxidant capacity measured by DPPH, ABTS, FRAP, oxygen radical absorbance capacity (ORAC), and cell antioxidant activity (CAA) methods. In general, polar solvents allow obtaining better antioxidant properties than the nonpoplar ones. However, considerable differences were observed even in the case of the application of solvents of similar polarity or the same solvent for the extraction of different types of propolis samples [31], pointing to the possible influence of other parameters as well as the impact of the molecular structure of solvents. Bittencourt et al. [31], for example, showed that that partition with dichloromethane enhanced the extraction of antioxidant compounds, especially in brown propolis, whereas partition with hexane significantly decreased their amount in green propolis extract (Table 1).

Importantly, despite numerous differences in its composition, propolis extract always possesses antioxidant properties. Even the aqueous extracts of propolis were shown to display antioxidant capacity in cell culture and animal studies [9, 12].

### 2.3. Antioxidant Effect of Propolis in Human Studies

Most of the studies regarding antioxidant properties of propolis have been performed on cell culture or animals. In the available literature, there are only a few studies investigating the antioxidant effect of propolis in humans.

Mujica et al. [44] evaluated the effects of the oral administration (twice daily, 15 drops each time, 90 days) of commercially available propolis solution (Beepolis®) on the oxidative status and lipid profile in a human population in Chile. The 90-day propolis supplementation resulted in a 67% decrease in the amount of thiobarbituric acid reactive substances (TBARS; lipid peroxidation derivative products) and 175% increase in reduced glutathione (GSH) level compared to the baseline. Net changes of both studied parameters were significantly higher in propolis supplemented group than those observed in the placebo group. Moreover, an increase in the HDL concentration on the 90th day of propolis supplementation compared to the baseline value was observed. The authors concluded that propolis supplementation appeared to have positive effects on oxidative status and the improvement of HDL and may thus reduce the risk of cardiovascular events.

Jasprica et al. [45], in turn, investigated the issue of the possible influence of 30-day supplementation with commercially available powdered propolis extract (a total daily dose of flavonoids was 48.75 mg) on antioxidant enzymes such as superoxide dismutase (SOD), glutathione peroxidase (GPx), catalase (CAT), and a lipid peroxidation marker—malondialdehyde (MDA)—in healthy individuals. In the male group, after 15 days of propolis treatment, a 23.2% decrease in MDA level was observed, whereas after 30 days, a 20.9% increase in SOD activity was found. Interestingly, MDA concentration in the end of treatment was similar to the baseline value. The propolis treatment had no effect on any of the studied parameters in women (*n* = 15). The authors concluded that the effect of propolis was both time and gender dependent and suggested a possibility of existence of only the transitory effect of propolis ingestion on lipid peroxidation.

The effect of Brazilian green propolis supplementation on antioxidant status in patients with type 2 diabetes mellitus (T2DM) was studied by Zhao et al. [46]. The propolis administration (900 mg/day, 18 weeks) was associated with an increase in serum levels of GSH and total polyphenols and reduction in serum carbonyls (protein oxidation markers) as well as lactate dehydrogenase activity. Moreover, the Brazilian green propolis group revealed a decreased TNF-*α* serum level and significantly increased IL-1*β* and IL-6 sera levels. However, the propolis treatment did not affect serum glucose, glycosylated hemoglobin, insulin, aldose reductase, or adiponectin. The above results indicate that propolis affects the oxidative stress in type 2 diabetic patients but not the parameters of diabetes.

### 2.4. Neuroprotective Effects of Propolis

Since mitochondrial damage and oxidative stress are critical events in neurodegeneration, in recent years, it has been suggested that antioxidant properties of the constituents of propolis may contribute to its neuroprotective effects. The effect of water-extracted brown propolis (WEBPs), from two regions of Iran, against cerebral ischemia-induced oxidative injury in a mouse model of stroke was studied by Bazmandegan et al. [9]. Regardless of the region of origin or the used doses, WEBP treatment resulted in a significant restoration of antioxidant enzymes activity and a decrease in both lipid peroxidation and the infarct volume, compared to the control group. Moreover, the treatment was associated with an improvement of neurological deficits measured with the Bederson scale and sensorimotor function measured with sticky removal tape test (Table 2). In another study performed on SH-SY5Y cells [47], it was found that the pretreatment with Brazilian green propolis reduced the H_2_O_2_-induced mitochondria-derived intracellular reactive oxygen species (ROS) generation as well as 8-oxo-2′-deoxyguanosine (8-oxo-dG, the DNA oxidative damage marker) immunofluorescence signal intensity. Propolis was also shown to increase the expression of the critical factors of synapse efficacy, brain-derived neurotrophic factor (BDNF), and activity-regulated cytoskeleton-associated protein (Arc). The obtained outcomes allowed the authors to suggest that propolis displays protective abilities against neurodegenerative damage, related to cognitive impairment caused by Alzheimer's disease or aging, via its antioxidant action (Table 2). This seems to be consistent with the results obtained by Nanaware et al. [48], who studied the neuroprotective activity of the macerated ethanolic extract of Indian propolis (MEEP) in rat model of Alzheimer's disease. MEEP significantly reversed the cognitive impairment of *β* amyloid-induced rats, which, among other things, was associated with increased antioxidant and decreased MDA levels. In addition, MEEP administration resulted in dose-dependent acetylcholinesterase inhibition, increased brain monoamine level, and improved memory deficits (assessed by increased BDNF level), which suggested that multiple mechanisms might be involved in that neuroprotective effect of propolis (Table 2).

The potential underlying mechanism of the neuroprotective effects of propolis or its compounds was studied by Jin et al. [6], Barros Silva et al. [7] and de Oliveira et al. [49].

Jin et al. [6] reported that pinocembrin, one of the most abundant flavonoids in propolis, inhibited 6-hydroxydopamine- (6-OHDA-) induced oxidative stress. Pinocembrin pretreatment induced the translocation of nuclear factor erythroid 2-related factor 2 (Nrf2) to the nucleus in a concentration- and time-dependent manner as well as the subsequent expression of antioxidant response element- (ARE-) mediated antioxidant genes encoding heme oxygenase-1 (HO-1) and *γ*-glutamylcysteine synthetase (*γ*-GCS). Nrf2 is known to play a key role in the adaptive response to oxidative and electrophilic stresses as well as maintaining the cellular self-defense. Under physiological condition, Nrf2 is localized in the cytosol and is associated with its negative regulator, Kelch-like ECH-associated protein 1 (Keap1). In response to oxidative/electrophilic stimuli, Nrf2 dissociates from Keap1 and translocates to the nucleus, where it forms a heterodimer with its obligatory partner Maf and then binds to the ARE sequence to activate transcription of genes encoding a large number of antioxidative and electrophile detoxification enzymes including HO-1 and *γ*-GCS. Pinocembrin also reduced the 6-OHDA-induced cell viability loss and apoptotic rate and partially inhibited the reduction of the Bcl-2 (an apoptosis inhibitor) to Bax (an apoptosis promoter) ratio following 6-OHDA treatment. The treatment of SH-SY5Y cells with the small interfering RNA (siRNA) directed against Nrf2 (Nrf2-siRNA) abolished pinocembrin-induced HO-1 and *γ*-GCS expression and its protective effects, which suggests that pinocembrin is protective against Parkinson's disease-related neurotoxin 6-OHDA through Nrf2/ARE pathway (Table 2). De Oliveira et al. [49] confirmed that pinocembrin exerted mitochondrial and cellular protection by the activation of the extracellular signal-regulated kinase 1/2-nuclear factor erythroid 2-related factor (Erk1/2-Nrf2) signaling pathway, since the inhibition of Erk1/2 or the silencing of Nrf2 abrogated these effects. Erk1/2 protein kinase is an activator of Nrf2. The researchers showed that pinocembrin pretreatment inhibited paraquat-induced lipid peroxidation, protein carbonylation, protein nitration, as well as the oxidation of thiol groups in the membranes of mitochondria of SH-SY5Y cells. Moreover, it activated the translocation of Nrf2 and increased the level of glutamate-cysteine ligase regulatory subunit (GCLM), glutamate-cysteine ligase catalytic subunit (GCLC), GSH, and HO-1. GCLM and GCLC are, respectively, regulatory and catalytic subunits of glutamate cysteine ligase—an enzyme which catalyzes the first and rate-limiting step in the production of the cellular antioxidant GSH. The above effects were blocked or inhibited with the Erk1/2 protein kinase inhibitor PD98059 or Nrf2 siRNA (Table 2).

The neuroprotective effect of another compound abundant in propolis, namely, caffeic acid phenethyl ester (CAPE), against 6-OHDA-induced dopaminergic neuronal loss in rats, was studied by Barros Silva et al. [7]. The cotreatment with CAPE decreased the hydrogen peroxide production in brain striatum homogenates. CAPE was also capable of scavenging ROS by neutralizing the unpaired electrons of DPPH but did not affect 4-hydroxy-2,2,6,6-tetramethylpiperidine-N-oxyl (TEMPOL, a stable nitroxyl antioxidant) in brain-affected areas. Additionally, CAPE protected against 6-OHDA-induced increase of metal levels (Cu, Fe, Mn, and Zn) as well as inhibited mitochondrial permeability transition (MPT), a mediator of neuronal death that triggers cytochrome c release and caspase-3 activation, and this effect was not associated with mitochondrial dysfunction. The authors concluded that basing on the obtained findings and its ability to cross the blood-brain barrier, CAPE could be a promising compound to treat Parkinson's and other neurodegenerative diseases (Table 2). Mahmoud et al. [50] demonstrated, in turn, that CAPE protected the brain against hexavalent chromium toxicity by preventing oxidative/nitrosative stress as well as modulating the JAK/STAT signaling pathway in rats. The researchers suggested that oxidative stress along with inflammation caused by Cr(VI) could directly activate the JAK/STAT signaling pathway in the cerebrum of rats, which was confirmed by increased JAK2 mRNA and protein expression and consequently STAT3 mRNA and protein phosphorylation in the cerebrum of Cr(VI)-induced rats. CAPE, in turn, by mitigating oxidative/nitrosative stress, downregulated JAK2/STAT3 signaling, which was also proved by a significant decrease in both JAK2 and STAT3 mRNA and protein levels in CAPE-treated group (Table 2).

### 2.5. Propolis Role in Mitigation of Chemotherapy Side Effect

In the literature, there are also studies aiming to determine propolis as a potential natural antioxidant to mitigate side effect of chemotherapy. Mitomycin C, cisplatin, and doxorubicin are recognised anticancer drugs used along with radiation or surgery. Unfortunately, their administration may cause diverse side effects, leading to considerable injury of organs and subsequently worsening life conditions. Some aspects of this harmful effect have been attributed to inducing oxidative damage.

Kumari et al. [4] showed that the hydroethanolic extract of Indian propolis (HEIP) displayed the protective effect against mitomycin C- (MMC-) induced genotoxicity and cytotoxicity which could be, at least partially, mediated via free radical-scavenging activity and inhibitory effect on lipid peroxidation. The potential geno- and cytotoxic effects of MMC in the bone marrow was manifested by a significant increase in the frequency of micronculeated cells and the percentage of apoptotic cells as well as the reduction in polychromatic erythrocyte (PCE) to normochromatic erythrocytes (NCE) ratio (P/N ratio) compared to the control group. However, MMC-induced toxic effects were significantly recovered by pretreatment with HEIP with the optimum dose being 400 mg/kg. In addition, HEIP possessed a considerable DPPH radical-scavenging activity (increasing along with an increase in HEIP concentration), and it exhibited almost such effectiveness as the standard use of ascorbic acid. HEIP was also found to possess the total antioxidant activity (evaluated by method based on the principle of reduction of molybdenum) and exhibited free radical-scavenging activity in FRAP measurement, but in this case, the results were not comparable with those obtained for ascorbic acid. Moreover, HEIP was shown to possess a substantial lipid peroxidation inhibitory activity, but again, it was also not as effective as the reference standard—Trolox (Table 2).

As chemotherapy is known to have fertility-related side effects, in the next study, Kumari et al. [51] investigated the effect of HEIP on MMC-induced testicular toxicity. The antioxidant effects of HEIP were assessed by measuring antioxidant/oxidant biomarkers in testicular tissue homogenate. MMC treatment resulted in long-term oxidative stress, whereas a single dose preadministration of HEIP was able to attenuate it to a certain degree—a significant decrease in MDA level and an insignificant elevation of GSH level and CAT activity were observed. MMC administration also led to a reduction in testicular function (testis weight, sperm count, sperm motility, and spermatozoa with normal head morphology) in a dose-dependent manner, which was alleviated by HEIP pretreatment (Table 2).

Alyane et al. [5], in turn, demonstrated that the propolis extract pretreatment substantially attenuated the peroxidative damage in the heart mitochondria following the injection of an acute dose of doxorubicin. Propolis led to a significant reduction in mitochondrial MDA formation and production of superoxide anion, as well as the restoration of respiratory control ratio (RCR—state III respiration/state IV respiration; indicates the tightness of the coupling between respiration and phosphorylation) and phosphate/oxygen ratio (P/O ratio; refers to the amount of ATP produced from the movement of two electrons through a defined electron transport chain. Additionally, a decreased rate and amplitude of mitochondrial swelling were observed (Table 2).

### 2.6. Propolis' Ability to Modulate Cardiovascular Disease Markers

The antioxidant properties of propolis have also been suggested to be responsible for its ability to modulate cardiovascular disease markers. Salmas et al. [52], for example, reported that oxidative alterations occurring in the kidney tissue of chronic hypertensive rats might be prevented via propolis, CAPE, as well as pollen administration. In the kidney tissue of N*ω*-nitro-L-arginine methyl ester- (L-NAME-) induced hypertensive rats, total antioxidant status (TAS) and paraoxonase (PON1, an important antioxidant preventing the oxidation of low-density lipoproteins) activity, were significantly decreased, whereas total oxidant status (TOS), asymmetric dimethylarginine (ADMA, an endogenous inhibitor of NO synthase), and nuclear factor kappa B (NF-*κ*B, regulated by intracellular redox state) were significantly increased. However, the coadministration of propolis, CAPE, and pollen restored all the disturbed parameters with the propolis samples being the most effective following by pollen and CAPE (Table 2).

Ahmed et al. [53], in turn, showed that Malaysian propolis (MP) pretreatment ameliorated the negative effects of isoproterenol-induced myocardial infarction in rats. MP exhibited high total antioxidant activity determined by both DPPH and FRAP assays. The isoproterenol administration resulted in significantly elevated lipid peroxides and reduced activities of cellular antioxidant defense enzymes in the myocardium. Moreover, it caused a significant increase in serum cardiac marker enzymes (creatinine kinase-MB, aspartate transaminase, lactate dehydrogenase, and alanine transaminase) and cardiac troponin I levels as well as altered serum lipid profiles. However, the pretreatment of ischemic rats with MP suppressed the above biochemical parameters as well as improved histopathological findings, suggesting the protective effect of MP against ISO-induced ischemia via its direct cytotoxic radical-scavenging activities and possibly via the inhibition of lipid peroxidation (Table 2).

The protective effect of six active compounds of Chinese propolis on H_2_O_2_-induced rat cardiomyocytes (H9c2) oxidative injury was also studied [54]. All tested compounds demonstrated significant cytoprotective activities; however CAPE, benzyl caffeate (BZC), and cinnamyl caffeate (CNC) exerted stronger effects than chrysin, pinobanksin, and 3,4-dimethoxycinnamic acid (DMCA). CAPE, BZC, and CHC increased H9c2 cellular antioxidant potential (by decreasing MDA level and increasing SOD and GPx activities), decreased intracellular calcium ion level, and prevented cell apoptosis (Table 2).

The protective effects of ethanol extract of propolis (EEP) against injury induced by oxidized low-density lipoprotein (ox-LDL) in human umbilical vein endothelial cells (HUVECs) were studied by Fang et al. [55]. An atherogenic role of ox-LDL in the progression of atherosclerotic cardiovascular disease is well known. EEP pretreatment ameliorated the ox-LDL-induced oxidative stress by reducing nicotinamide adenine dinucleotide phosphate (NADPH) oxidase activation, ROS, and MDA generation as well as elevating antioxidant enzyme activities. In addition, EEP reduced ox-LDL uptake by HUVECs and attenuated ox-LDL-upregulated expression of lectin-like oxidized low-density lipoprotein receptor-1 (LOX-1—a critical molecule responsible for ox-LDL uptake by endothelial cells) both at the mRNA and at protein levels [56]. Moreover, EEP in a dose-dependent manner protected against the decrease in cell viability as well as the increase in lactate dehydrogenase (LDH) release, caspase-3 activation, and apoptosis induced by ox-LDL. The obtained outcomes allowed the authors to conclude that EEP appeared to protect HUVECs from ox-LDL-induced injury via, at least partially, the modulation of LOX-1-mediated oxidative stress.

Tian et al. [57] proved that the ethanol extract of propolis might protect macrophages from ox-LDL-induced apoptosis and the underlying mechanism at least partially involved its ability to suppress the CD36-mediated ox-LDL intake and the subsequent activation of the endoplasmic reticulum (ER) stress-C/EBP homologous protein (CHOP) pathway; it significantly suppressed the phosphorylation of double-stranded RNA-activated protein kinase-like ER kinase (PERK) and eukaryotic translation initiation factor 2*α* (eIF2*α*) as well as the upregulation of glucose regulated protein 78 (GRP78) and the proapoptotic protein CHOP.

El-Awady et al. [58], in turn, reported that propolis could protect against high glucose-induced vascular endothelial dysfunction via decreasing oxidative stress in isolated rat aorta. Incubation of aortic rings with propolis extract prevented high glucose-induced the impairment of phenylephrine-induced contraction and acetylcholine-induced relaxation. Additionally, a SOD activity and GSH concentration increase as well as a decrease in MDA level were observed (Table 2).

### 2.7. Propolis as the Protective Agent against Prooxidants' Toxicity

Antioxidant properties of propolis encouraged the research concerning its application as an agent preventing or alleviating harmful oxidative processes caused by various factors, like trichlorfon, tebuconazole, paracetamol, methylmercury, or UV irradiation.

The beneficial effect of propolis on trichlorfon-induced prooxidant/antioxidant and haematological parameters alterations in carp *Cyprinus carpio* was stated [59]. Fish were exposed to sublethal concentrations of trichlorfon—a toxic pesticide commonly used in aquaculture to eliminate fish parasites, and propolis was administered simultaneously. The treatment with propolis caused the alleviation of trichlorfon-induced negative alterations in the haematological parameters (red and white blood cell counts, haemoglobin concentration, haematocrit, erythrocyte indices, mean corpuscular volume, mean corpuscular haemoglobin, and mean corpuscular haemoglobin concentration) as well as oxidant markers (MDA, GSH, SOD, CAT, and GPx) in the liver, kidney, and gill samples (Table 2). Ferreira et al. [12], in turn, showed that bee products, such as propolis, bee pollen, royal jelly, and honey, prevented and/or reversed tissue (brain, liver, and kidney) oxidative damage induced by tebuconazole (an agrochemical fungicide) in fish by increasing the enzymatic activities of SOD, CAT, and glutathione-S-transferase (GST) and decreasing lipid peroxidation (Table 2).

Aksu et al. [60] investigated the effect of chrysin (CR, a flavonoid occurring in propolis) pretreatment against paracetamol- (PRC-) induced reproductive toxicity in male. The treatment with PRC resulted in reduced sperm motility, antioxidant enzymes activity (SOD, CAT, and GPx), and GSH level, as well as increased dead sperm rate, abnormal sperm cell rate, apoptosis, and MDA level in testicular tissue. CR was found to mitigate the above effects in a dose-dependent manner with the higher dose being more effective. The authors concluded that the possible protection mechanism might be dependent on the antioxidant activity of CR (Table 2). A protective effect of chrysin against methylmercury-induced genotoxicity and oxidative stress was also studied by Manzolli et al. [61]. The cotreatment with chrysin resulted in the restoration of GSH level, and it decreased the formation of comets in leukocytes and hepatocytes (Table 2).

Saito et al. [62], in turn, demonstrated that Brazilian green propolis and its three main constituents (3,5-di-*O*-caffeoylquinic acid, 3,4-di-*O*-caffeoylquinic acid, and chlorogenic acid) increased the HO-1 (heme oxygenase 1) expression and accelerated Nrf2 nuclear translocation after UVA irradiation (the major cause of human skin aging) in human skin fibroblast cells (NB1-RGB).

Propolis has also been suggested to be useful for improving wound healing, which is possibly owed to its antioxidant activity (Table 2). Cao et al. [63] investigated the protective effects of the ethanol extract of Chinese propolis (EECP) against H_2_O_2_-induced oxidative stress in mouse L929 fibroblast cell lines. EECP not only showed significant protective effects against H_2_O_2_-stimulated L929 cell death but also reduced the decline of collagen mRNA expression in a significant way. Moreover, EECP induced the expression of antioxidant-related genes, such as *HO-1* (encoding heme oxygenase 1), *GCLM* (encoding glutamate-cysteine ligase regulatory subunit), and *GCLC* (encoding glutamate-cysteine ligase catalytic subunit) at both mRNA and protein levels in skin fibroblasts (Table 2). Heme oxygenase-1 breaks down heme to carbon monoxide, iron ions, and biliverdin, which is subsequently reduced to bilirubin. Both biliverdin and bilirubin are potent antioxidant agents.

Arabameri et al. [64] proved that the Iranian propolis could significantly prevent oxidative stress (by alleviating the changes in ferric-reducing antioxidant power, SOD, GPx, and MDA) as well as histopathological changes (the number of ovarian follicles, oocytes, and oocytes diameter) in the ovaries of the neonatal rat following maternal separation stress (infants were separated from their mothers 6 hours per day). All three applied doses exerted positive effect, but the most effective was the dose of 200 mg/kg (Table 2). Mişe Yonar et al. [65], in turn, investigated the effect of dietary propolis on the number and size of pleopodal egg and oxidative/antioxidant status of freshwater crayfish. Dietary propolis supplementation resulted in a significant decrease in MDA level and CAT and GPx activities as well as a significant increase in SOD activity in hepatopancreas and ovarium. The pleopodal egg number produced per gram of the body weight and total pleopodal egg number significantly increased, whereas the pleopodal egg size significantly decreased following propolis administration. The authors suggested that the reduced activity of CAT and GSH-Px following propolis supplementation could have resulted from the inhibition of superoxide radical formation by the dietary propolis and concluded that propolis improved efficiency in the crayfish and reduced the oxidative stress under controlled hatchery conditions.

Zhang et al. [66] demonstrated that ethanol extracts of Chinese propolis (EECP) could reduce the intracellular ROS level not only in the H_2_O_2_-induced RAW264.7 cells but also in normal RAW264.7 cell (not subjected to any factor). This suggested that propolis could be capable of reducing oxidative stress generated not only under pathological but also under physiological conditions. Similar to Cao et al. [63], the authors found that EECP in a time- and dose-dependent manner elevated the expression of antioxidant genes such as HO-1, GCLM, and thioredoxin reductase 1 (TrxR1) on both the mRNA and protein levels (Table 2). TrxR, along with NADPH and thioredoxin, is a component of thioredoxin (Trx) system that creates a key antioxidant system as defense against oxidative stress through the disulfide reductase activity, regulating protein dithiol/disulfide balance [67]. Since EECP also increased expressions of phosphorylated Erk and the nucleus translocation of Nrf2, the researchers suggested that propolis might modulate the expression of HO-1, TrxR1, and GCLM via Erk kinase/Nrf2 signal pathway.

### 2.8. Propolis as a Cosmetics Additive

Propolis was also studied with regard to its potential application in cosmetics. The research revealed that it can act as a sunscreen agent [68] and could be used as an ingredient of sunscreen cosmetics [69]. Gismondi et al. [70] studied its usage as an agent-protecting essential oil, added to sunscreens with the aim of preventing cytotoxic and proradical effects of their components, against damage caused by UV radiation. *Lavandula angustifolia* Miller essential oil samples, pure or added with 30% ethanol propolis solution at a dose of 1%, were subjected to UV radiation. UV exposure depleted the antioxidant activity of essential oil (DPPH, ABTS, and FRAP assay). Propolis supplementation not only prevented this effect but also considerably increased this parameter in both exposed and nonexposed samples. Those promising results were confirmed by experiment performed on highly metastatic murine B16-F10 melanoma cells. The addition of essential oil samples to culture media caused an increase in cellular GPx, SOD, and CAT activity, but in the case of UV-exposed one, this effect was much less or even slight. However, additional propolis prevented the deterioration of oil properties by UV radiation,as in this case, the results obtained in both oil+propolis+UV and oil+propolis treatments were generally not lower that in the case of essential oil alone.

## 3. Bee Pollen

Bee pollen is produced from plant flower pollen, which is collected by bees and mixed with nectar or the salivary gland secretion of the insects. In such form, it is transported, placed on the hind legs, to hives. Then flightless bees mix it with their saliva and pack into honeycombs, covered with a mixture of wax and honey. Under such conditions, the anaerobic fermentation proceeds with the formation of lactic acid, serving as a preservative. The substance, produced in this way, makes a source of nutrients for both adult bees and larvae. The beekeepers collect bee pollen using traps that enable to separate pellets from insects' legs [71–74].

Pollen food energy is rather high; for instance, bee pollen produced by *Apis mellifera*, collected in Thailand and containing mainly corn pollen, showed its value to be as much as 397.16 kcal/100 g [42]. In 22 samples of bee pollens collected in Portugal, the obtained values ranged from 396.4 to 411.1 kcal/100 g [75]. It is even named “only perfectly complete food” [75] or “the life-giving dust” [76].

### 3.1. Bee Pollen Composition

The components of bee pollen comprise of a great number of different substances including nutrients (proteins, carbohydrates, and lipids), amino acids (bee pollen is a rich source of leucine, isoleucine, and valine—branched, exogenous amino acids), fatty acids and their esters, vitamins (carotenoids, B, E, H, and folic acid), minerals (macro- and microelements), as well as phenolic organic compounds—flavonoids, phenolic acids, and their derivatives [2, 15, 42, 75, 77–83]. Additionally, the presence of different organic acids (oxalic, tartaric, malic, citric, succinic, acetic, lactic, and gluconic) was found, the latter exhibiting the highest concentration. Among inorganic components macroelements (sodium, potassium, calcium, and magnesium), microelements (iron, zinc, manganese, and copper) as well as some other metals (chromium, aluminium, strontium, tin, nickel, and vanadium) were detected. The content differed considerably in some cases, depending on the source region and plants, particularly as for gluconic acid, potassium, calcium, iron, manganese, and zinc [79].

Numerous studies concerning bee pollen included the determination of their detailed composition, particularly considering the biologically active compounds. The following flavonoids and their derivatives were identified as components of rape (*Brassica campestris* L.) bee pollen: quercetin, naringenin, kaempferol, and isorhamnetin as well as rutin and 3-*O*-glucosides of quercetin and kaempferol [83]. In the bee pollen from *Cistus* sp. of Spanish origin, in turn, kaempferol, kaempferol-3-glucoside, quercitin, quercetin-7-rhamnoside, and isorhamnetin were found [43], whereas in Croatian *Cystus incanus* L. bee pollen, galangin, kaempferol, chrysin, and pinocembrin were detected [78]. In some samples, the presence of herbacetin, myricetin, tricetin, luteolin, and 3-*O*-methylquercetin was proved [84]. In Egyptian bee pollen, quercetin, rutin, catechin, epicatechin, kaempferol, apigenin, naringenin, and luteolin were identified [74]. The glucosides of the following anthocyanins, delphinidin, petunidinm, and malvidin were found in bee pollen collected in Spain [85]. According to Silva et al. the analysis of pollen loads collected by stingless bees *Melipona rufiventris* revealed the presence of different flavonoids, including dihydroquercetin, luteolin, quercetin, isorhamnetin, and isorhamnetin glucoside (isorhamnetin-3-*O*-(6″-*O*-E-*p*-coumaroyl)-*β*-D-glucopyranoside), which was detected in bee pollen for the first time [41].

As for phenolic acids on average, they constitute up to 0.19% of bee pollen and comprise of the derivatives of benzoic, cinnamic, and phenylacetic acids. Gallic acid (3,4,5-trihydroxybenzoic acid) is characterized by a great antioxidant activity [15].

Among the constituents of bee pollen, the following phenolic acids and their esters were reported to be identified: benzoic acid derivatives—*p*-hydroxybenzoic acid, gallic acid, syringic acid, vanilic acid, and protocatechuic acid—as well as cinnamic acid derivatives—*p*-coumaric acid, ferulic acid, caffeic acid, and their glycerol esters. Other more complicated derivatives like rosmarinic acid dihexoside as well as amide derivatives of hydroxycinnamic and ferulic acids were also found [14, 41, 71, 74, 77, 86].

### 3.2. Relationships between the Antioxidant Capacity of Bee Pollen and Its Composition

Bee pollen antioxidant properties have been investigated in many *in vitro* studies using DPPH, ABTS^+^, and FRAP methods. It is well known that the antioxidant capacity of bee pollen is dependent on its content. But the numerous studies, which have been carried out with the aim of determining the composition and properties of different bee pollen samples, proved a considerable diversity of the obtained results. Some studies have shown a strong positive correlation between the total content of phenolic compounds and antioxidant capacity of bee pollen [79, 81], whereas others found no considerable relationships [71]. In the next study [87], in turn, not phenolic compounds but phenylpropanoid content was found to be correlated with the total antioxidant activity measured by the inhibition of linoleic acid peroxidation. According to Sousa et al. [85], in turn, flavonols may act as both antioxidants and prooxidants in their reduced and oxidized forms, respectively, whereas anthocyanins act as prooxidants. It has also been found that both content and properties of bee pollen are dependent on the kind of its plant source as well as the conditions of the plants growing like soil or climate. The time of harvesting creates an additional factor affecting these properties [2, 75, 76, 79, 80]. The potential influence of some kind of treatment (freezing as well as freezing and subsequent dehydratation) on the content and properties of bee pollen was also reported. While the chemical composition was not affected by freezing or freezing and subsequent drying, the antioxidant activity was enhanced by freezing and additional drying. The researchers attributed the observed effects to a moisture decrease, leading to antioxidants concentration [71]. The differences in polyphenolic compounds, in total as well as particular types, were also found, showing considerable dispersion, for example, LeBlanc et al. [11] reported that mimosa bee pollen contained 34.85 mg/g of polyphenolics expressed as gallic acid equivalents, while in the yucca one, only 19.48 mg/g, and in the palm one, 15.91 mg/g were found. The flavonol content in *Pyrus communis* bee pollen was found to be 1349 mg/100 g, but in the *Lamium purpureum* one, it reached only 171 g/100 g [87]. In different samples of bee pollen collected in northeastern Brazil in the period of nine months (January–November), flavonoid profiles were found to differ, depending on the time of harvesting and the predominant pollen type [81].

Similar to the case of propolis, the research has shown that the type of the extraction solvent used may affect the properties of the pollen extract to a considerable degree (Table 1). This is connected with different solubilities of particular components of bee pollen in solvents of diverse polarities. It was proved that the application of nonpolar solvents resulted in extracts of very low antioxidant activity, whereas the polar ones allowed obtaining the better antioxidant properties. However, considerable differences were observed even in the case of the application of solvents of similar polarity [11, 41, 42]. The optimal condition for bee pollen extraction was studied by Kim et al. [13]. In their experiment, the total extract obtained by extraction with 80% methanol (twice) was then successively partitioned using solvents of different polarities: n-hexane, dichloromethane, ethyl acetate, and n-butanol. Ethyl acetate and n-butanol fractions exhibited the greatest activity, so in the next step, the optimal conditions of extraction were estimated by response surface methodology using the Box-Behnken design (BBD) with three-level three-factor. The variables were ethyl acetate concentration in methanol, temperature, and time. The solvent concentration proved to exhibit the greatest impact, and the optimal parameters were established as follows: 69.6% ethyl acetate in methanol, 10.0°C and 24.2 h. The calculated values were confirmed experimentally, as the extract obtained under the theoretically estimated conditions showed the antioxidant activity and tyrosinase inhibition very similar to those predicted by statistical methods [13]. The dependence of extract properties on the used extraction solvent was also confirmed in the animal research. In rats with induced hind paw edema, the oral administration of bee pollen bulk showed mild suppressing properties, and water extract had practically no effect, while ethanol extract displayed the greatest effectiveness [43].

### 3.3. Bee Pollen Role in Mitigation of Chemotherapy Side Effect

Bee pollen has been found both to alleviate the deterioration of antioxidant barrier and instead of nad to inhibit lipid peroxidation process following chemotherapy.

Huang et al. [88] found that the intraperitoneal treatment with cisplatin resulted in the extensive deterioration of liver and kidney functions. This harmful effect involved a significant increase in the concentration of a lipid peroxidation marker MDA and iNOS as well as a well-marked depletion of activities of chosen antioxidant enzymes. However, the additional intragastrical pretreatment with *Schisandra chinensis* bee pollen was able to alleviate these effects in a dose-dependent way. The additional evidence for the antioxidant influence of bee pollen was the comparison of the described results with the effects observed in animals treated according to the same design with cisplatin and an acknowledged antioxidant vitamin—ascorbic acid—whose impact was comparable with that observed for bee pollen (Table 3). The relationships between antioxidant properties and protective influence of bee pollen against cisplatin were also confirmed by Tohamy et al. [89]. In a study performed on cisplatin-exposed male mice, distinct symptoms of oxidative stress in organs (kidney, liver, and testis) were observed as the lipid peroxidation process was greatly intensified, while CAT activity and GSH concentration markedly depressed. However, the oral coadministration of Egyptian bee pollen water extract significantly alleviated prooxidant changes. Moreover, bee pollen alone decreased neither CAT nor GSH and distinctly inhibited lipid peroxidation in the kidney and testes. As the studied organs show a great vulnerability to the toxic action of cisplatin, the obtained results made the authors suggest the potential effectiveness of bee pollen at alleviating cisplatin-induced side effects. It seems to be worth noticing that in this case, the bee pollen proved to be effective even when administered after cisplatin application. The experiment performed by scientists from Malaysia supported the abovementioned findings. According to the authors, the methanol extract of bee pollen of Malaysian stingless bee (*Lepidotrigona terminata* (*L. terminata*)), displaying a distinct, dose-dependent antioxidant activity, was also effective for the antiproliferation of cells. Such an effect was observed in the case of both cancer (MCF-7) and normal (L929) ones with the IC_50_ value being much lower for cancer cells. Then the effect of cisplatin alone and in cotreatment with bee pollen extract was studied on MCF-7 cells. Cell proliferation was distinctly inhibited by cisplatin, and additional bee pollen revealed a potentializing influence on cisplatin action. In the next stage, the analysis of the influence of bee pollen and cisplatin combination on MCF-7 cell line was performed using CompuSyn software to evaluate if these agents work in an antagonistic, synergic, or additive way. The statistical analysis allowed the authors to suggest that these two substances acted in synergistic way. In conclusion, the researchers suggested the possibility of the application of the studied bee pollen, capable of aiming at potentiating the effectiveness of the therapy and allowing the decrease of the dose of chemoprotective drugs [90] (Table 3).

### 3.4. Bee Pollen as a Protective Agent against Prooxidants' Toxicity

Antioxidant properties of bee pollen encouraged the research concerning its application as an agent preventing or alleviating harmful oxidative processes occurring in organisms or caused by different factors. The performed studies included interesting, diverse issues and resulted in considerably promising findings.

Turkish scientists investigated bee pollen as a protective agent against carbon tetrachloride hepatotoxicity. The results were additionally compared with those obtained for silibinin, an active component of silymarin (a plant-origin substance used in hepatic disorder cure). All the applied treatments caused a decrease in body weight gain. However, this effect was the least in the case of the coadministration of CCl_4_ and the higher dose of bee pollen. A significant enhancement of liver injury markers—plasma activity of transferases ALT and AST observed in CCl_4_-exposed rats—was considerably alleviated in animals cotreated with both silibinin and bee pollen. Furthermore, the effect of the higher bee pollen dose was not markedly different from that observed in the case of silibinin. Liver and plasma MDA were found to be significantly increased by CCl_4_ exposure, and again, both studied protective agents were capable of reversing this effect, although that in liver silibinin was more effective. The SOD activity in plasma, RBC, and liver was depressed in CCl_4_-exposed rats, and in this case, both silibinin and bee pollen proved to lack protective influence. As chestnut bee pollen was found to contain antioxidants and a substantial antioxidant capacity measured by FRAP and DPPH methods, the authors suggested the possibility of replacing silibinin by bee pollen in liver disorders cure, all the more because the harmful effects were observed in silibinin-treated animals—decrease in body weight gain, severe diarrhea, and, consequently, mortality [1] (Table 3).

Bee pollen was also shown to alleviate aflatoxin–induced oxidative processes in spleen by a decrease in the H_2_O_2_ level accompanied by GSH enhancement and NO proper generation [91].

In another animal study, bee pollen was investigated with regard to its possible application as an agent alleviating stress induced by exercise. Taking into account the fact that nutrients contained in bee pollen are barely absorbed in the gastrointestinal track due to the tough coat enclosing the components inside and making digestion difficult to proceed, the authors carried out a very interesting comparison of the neat and processed monofloral Indian mustard bee pollen. The processed one was obtained by mixing with an edible lipid-surfactant mixture (Captex 355 and Tween 80 in different ratios). The composition of the lipid-surfactant mixture influenced the total polyphenol content in the obtained processed samples, with the ratio of 1 mg of bee pollen : 500 mg of Captex 355 : 750 mg of Tween 80 showing the highest value. For this reason, that sample was chosen for further studying using an animal model as a protective agent against oxidative changes caused by exercise. In rats subjected to chronic exercise, the distinct evidence of oxidative stress was shown as SOD, and GSH were found to be markedly decreased, while MDA and NO significantly increased in the gastrocnemius muscle. Additionally, exercised animals displayed significantly decreased body and gastrocnemius muscle weights compared to the control. All these exercise-induced changes were partially reversed by oral treatment with both neat and processed bee pollen in a dose-dependent way, and the processed one proved to have much greater efficiency. Furthermore, the processed bee pollen, given alone to nonexercised animals, generally did not substantially affect the studied parameters in comparison with the control. The authors concluded that processing improved the availability of bee pollen nutrients and subsequently all beneficial effects [92] (Table 3).

Bee pollen has also been found to show some protective effect against oxidative damage observed in fish environmentally exposed to tebuconazole (a fungicide of high toxicity to aquatic organisms), but the obtained results were not unambiguous. Tebuconazole caused a considerable intensification of lipid peroxidation in chosen organs and a decrease in liver SOD. Additional bee pollen considerably reversed these effects. However, in the case of CAT, the obtained results were not so promising. Tebuconazole alone enhanced its activity in the liver and brain and showed no significant effect in kidney. The cotreatment with bee pollen displayed diverse effects, depending on dose and organ, with no determined tendency. But the most important fact was a significant CAT decrease versus control observed in the kidney and brain of fish exposed to higher concentrations of bee pollen. Furthermore, bee pollen alone also depressed CAT in those organs compared to control [12].

Almaraz-Abarca et al. [93] investigated the properties of bee pollen from mesquite (*Prosopis juliflora*) collected in Mexico and also received inconsistent results. Bee pollen extracts of two flavonol concentrations prevented lipid peroxidation observed in the liver of mice exposed to bromobenzene but the results reached statistical significance only in the case of higher dose. However, the extract of higher concentration given alone caused a significant intensification of liver lipid peroxidation in mouse, comparably with the one observed in bromobenzene-treated animals. Interestingly, the extract of lower concentration showed a great antioxidant effect as lipid peroxidation in this case was even decreased when compared to the control with no treatment. The authors concluded that in the absence of any oxidative stress-inducing factor, the administration of the high concentration of flavonols itself may induce oxidative damage occurrence. According to the authors, the confirmation of such an assumption could be the reports revealing that both polyphenols and an acknowledged antioxidant vitamin C may act as prooxidants in the presence of transient metal ions. Despite the similarity of the effects, the authors do not postulate the similarity of the mechanism of prooxidant influence of flavonoids and vitamin C. However, these outcomes show the necessity of proper precaution in the application of bee pollen, particularly in considering the dose (Table 3).

Similar conclusions were drawn from the experiment performed by Sousa et al. [85]. Studying bee pollen from *Echium plantagineum* L., they used three different substances: its extract enriched in flavonols (fraction I), its extract enriched in anthocyanins (fraction II), and the combination of both extracts (the whole extract). Both I and II extracts contained kaempferol glucosides, and additionally in fraction II anthocyanins, glucosides of delphinidin, petunidin, and malvadin were detected. All three extracts were studied *in vitro* considering their influence on viability, reactive species, and antioxidants in Caco-2 cells. Interestingly, the extracts exhibited varied activity under different conditions. As for cells not subjected to any factor, neither fraction I nor fraction II caused any changes in cell viability measured by MTT assay, while the whole extract containing the combination of flavonols and anthocyanins, used in high concentration (20 mg/mL), caused a significant cellular viability depression. The effect of pretreatment with bee pollen extracts on the viability of cells induced by butyl hydroperoxide (*t*-BHP), whose presence stimulated reactive oxygen species production, was also evaluated. Herein, fraction II used in higher concentrations (2.5–20 mg/mL) and the whole extract (2.5–10 mg/mL) intensified *t*-BHP-induced harmful effect, while the highest concentrations of fraction I showed some insignificant but distinct protective effect. Additionally, the percentage values of cell viability, obtained for the whole extract, applied in concentration of 20 mg/mL, were the same, regardless of *t*-BHP presence or absence. These outcomes allowed the authors to suggest that anthocyanins acted as prooxidants, while flavonols supported antioxidant barrier but, in their oxidized forms, might also contribute to prooxidant processes. Lower concentrations of fractions I and II were also efficient at decreasing reactive species level. Interestingly, high concentration (20 mg/mL) prevented reactive species production only after a short *t*-BHP exposure. Along with the exposure, lengthening fraction II was proved to be ineffective, while fraction I began to act as a prooxidant as reactive species generation was shown to be enhanced. The whole extract did not show a significant antioxidant efficacy, and the higher concentrations exerted even prooxidant effect, particularly after a longer time of *t*-BHP exposure. Next, reduced glutathione was determined in the cells treated with different concentrations of the studied extracts, with and without subsequent exposure to *t*-BHP. The only effects were observed in cells exposed to *t*-BHP—a significant GSH increase after pretreatment with 20 mg/mL of the whole extract and a slight enhancement after using higher doses of fraction I. The authors tried to explain those complex observations pointing to GSH participation in *t*-BHP defusing, the bioactivation of *t*-BHP by cytochrome P450 as well as the inhibition of the influence of flavonoids (mainly anthocyanins) on cytochrome P450 enzymatic activity, the capacity of anthocyanins for being transformed into radicals, as well as the ability of *t*-BHP to form species more active than those produced by the process of its biotransformation. Concluding, the authors underlined the necessity of taking the proper precautions in using bee pollen, particularly considering the dose.

### 3.5. Bee Pollen in Cosmetics

The beneficial influence of bee pollen on skin condition has been known from ancient times. Recently, as the return to natural medicine agents is being observed, some studies have been carried out with the aim of clarifying the mechanism of these effects.

Sun et al. [86], in their experimental study, confirmed the possibility of bee pollen using in skin therapy, connected with its antioxidant ability. Chinese scientists studied composition, antioxidant activity, as well as its influence on the melanogenesis of two rape bee pollen extracts containing free or bound phenolics. Both extracts exhibited antioxidant capacity measured by DPPH, ABTS, and FRAP methods, although the free phenolic one proved to be much more effective as an antioxidant. In the next stage of the study, the effect on melanin synthesis was investigated. Tyrosinase activity was inhibited in a dose-dependent way by both extracts with the free one being more effective. Since some phenolic compounds also showed the same effect, the authors assumed the existence of some relationship between phenolic profile and inhibitory influence on tyrosinase activity. The tyrosinase inhibition was studied considering the fact that melatonin, despite its protective role for the skin, in excessive amounts, may itself exert harmful effects like reactive oxygen species generation and pigmentation. The effect of free phenolic extract of bee pollen on melanogenesis was also studied using B16 mouse melanoma cells. The studied substance decreased intracellular tyrosinase activity and melanin relative content in a very distinct, dose-dependent way. Moreover, taking into account the connection between the melanogenesis and generation of oxygen reactive species and consequently the role of intracellular reducing activity in melanogenesis regulation, GSH/GSSG value was measured. Free phenolic bee pollen extract again showed a high effectiveness at increasing reducing power by enhancing intracellular GSH/GSSG ratio, indirectly contributing to the depression of melanin synthesis. All the presented results point to the usefulness of bee pollen in protecting the cell against abnormal melanogenesis, which cannot be overrated as melanin is responsible for numerous skin disorders, from freckles up to malignant melanoma [86]. The usefulness of bee pollen in manufacturing cosmetics protecting the skin against hyperpigmentation and oxidative stress was also confirmed by Korean scientists [13].

## 4. Royal Jelly

Royal jelly is a secretion from mandibular and hypopharyngeal glands of young bees of the *Apis mellifera* species [94]. This is a white or yellowish cream substance that makes food for young bee larvae (but no longer than three days, and then they are fed with a mixture of pollen, nectar, and honey) and the only food for the queen in both the larval and adult stages [95–97]. This difference in the way of feeding is considered as the main factor responsible for the differentiation in the development of bee workers and the queen. In comparison to the workers' food, the royal jelly contains less water and four times more sugars, more proteins, and different concentrations of some mineral salts [96]. This unique composition of royal jelly leads to changes in gene expression (most probably through epigenetic mechanisms) which allows, for instance, full ovarian development to proceed [97]. Thanks to royal jelly, the queen could live up to five years (workers usually live about 45 days) and lay about 2000–3000 eggs a day [98]. For commercial use, royal jelly is collected from the queen's cells, as they are the richest sources of this product—it is produced in a much larger amount than queen larvae are able to consume [94]. According to some sources, the annual production of royal jelly amounts to several thousand tons—about 2000 tons are produced only in China [99].

### 4.1. Royal Jelly Composition

From the chemical point of view, the royal jelly is an emulsion of proteins, sugars, and lipids in water. Moreover, it contains about 1.5% mineral salts (mainly copper, zinc, iron, calcium, manganese, potassium, and sodium salts) and small amounts of flavonoids, polyphenols, and vitamins (biotin, folic acid, inositol, niacin, pantothenic acid, riboflavin, thiamine, and vitamin E) [95, 99–101]. Among RJ, flavonoids can be distinguished: flavanones (hesperetin, isosakuranetin, and naringenin), flavones (acacetin, apigenin, and its glucoside, chrysin, and luteolin glucoside), flavonols (isorhamnetin and kaempferol glucosides), and isoflavonoids (coumestrol, formononetin, and genistein) [102].

The water content in the royal jelly is 50–70% [23, 95]. The total sugar content fluctuates between 7 and 21.2% and mainly consists of fructose and glucose [95, 99, 103]. Fructose and glucose together account for 90% of all sugars [99, 104]. Sucrose is always present but often in variable concentrations (2.86% according to Kanelis et al. [99], 2.1% according to Kolayli et al. [23], 0.5–2% according to Oršolić [105], and 0.2% according to Wytrychowski [106]). Studies have also revealed the presence of other oligosaccharides, such as trehalose, maltose, gentiobiose, isomaltose, raffinose, erlose, and melezitose [103, 104].

The total protein content in the royal jelly, according to different researchers, varies between 8 and 9% [23, 104, 105]. The electrophoretic analysis of royal jelly from two bee subspecies—*Apis cerana japonica* and *Apis mellifera*—revealed 21 different bands of proteins on the gel of which 14 bands were common to both subspecies [107]. The so-called major royal jelly proteins (MRJPs) represent about 90% of the total protein content [108]. Interestingly, according to Kamakura [109], the ability of the royal jelly to modulate the development of female larvae may be partially related to the presence the most abundant protein—major royal jelly protein 1 (MRJP1). Silici et al. [100] pointed out that very important components of the royal jelly are free amino acids. Using the LC/MS method, they indicated that it contained amino acids such as lysine (the biggest amount—62.43 mg/100 g); proline (58.76 mg/100 g); cystine (21.76 mg/100 g); aspartic acid (17.33 mg/100 g); and less than 5 mg/100 g of valine, glutamic acid, serine, glycine, cysteine, threonine, alanine, tyrosine, phenylalanine, hydroxyproline, leucine-isoleucine, and glutamine. According to these scientists, the antioxidant activity of royal jelly may be related to the biological effect of free amino acids.

The total content of fats and fatty acids in the royal jelly is estimated to be in the range of 7–18% [95, 104]. Instead of carboxylic acids with 14–20 carbon atoms commonly found in animals and plants, the royal jelly contains short hydroxy fatty acids with 8–12 carbon atoms in the chain and dicarboxylic acids. About 80–90% of the fatty substance fraction is an extremely rare free fatty acid with an unusual structure. The major fatty acid is 10-hydroxydecanoic acid (10-HDA), whose presence has not been reported in any other natural raw material or even in any other product of apiculture [23]. According to different investigators, the content of 10-HDA in the royal jelly ranges from 0.75 to 3.39% [99]. It is noteworthy that this acid is considered as one of the most important components from which the royal jelly biological activity derives. Other carboxylic acids are 10-hydroxy-2-decenoic acid (10H2DA) and sebacic acid (SA) (Figure 4) [24].

Considering the antioxidant activity, very important ingredients of the royal jelly are flavonoids and phenolic compounds. According to Nabas et al. [104], the royal jelly contains 23.3 ± 0.92 GAE *μ*g/mg total of phenolics and 1.28 ± 0.09 RE *μ*g/mg of total flavonoids. Interestingly, Liu et al. [110] found higher contents of polyphenolic compounds (and also proteins) in the royal jelly harvested 24 hours than in that harvested 48 or 72 hours after collection from the larvae. Hence, the authors suggest that the harvesting time of royal jelly can affect the content of antioxidant compounds and, thus, the therapeutic potential of the product. The GC/MS analysis performed by Kanbur et al. [111] showed that the main phenolic compounds contained in the royal jelly were pinobanksin as well as organic acids and their esters, for example, octanoic acids, 2-hexenedioic acid and its esters, dodecanoic acid and its ester, 1,2-benzenedicarboxylic acid, and benzoic acid.

### 4.2. Royal Jelly as a Scavenger of Free Radicals

In the available data, there are some reports confirming the role of royal jelly as a scavenger of free radicals [104, 110, 112, 113]. For instance, Liu et al. [110] investigated the antioxidant properties of the royal jelly expressed as the radical-scavenging effect upon DPPH, hydroxyl, and superoxide radicals. The researchers also evaluated its reducing power, inhibition effect upon linoleic acid oxidation, and superoxide dismutase activity. The obtained results were compared depending on the larval age (1-, 2-, or 3-day old) and time of harvest after the larval transfer from the queen cell cups to the bee hives (24, 48, and 72 h). The authors noted DPPH radical-scavenging effect (in the range of 43.0–62.8%) as well as the inhibitory effect on the superoxide radical formation (ranging from 23.9 to 37.4%) and on hydroxyl radical formation (48–68%). Moreover, the royal jelly sample demonstrated an inhibitory effect on linoleic acid peroxidation (8.6–27.9%). In all cases, the strongest scavenging effect of RJ was noted in the samples taken from the youngest larvae (1 day old) transferred into bee hives for the shortest time (24 h). In addition, the same royal jelly samples proved to have the strongest reducing power. On the other hand, the SOD activity of the royal jelly collected at 72 h after larval transferring of 3-day old larvae was significantly higher than that of the others. Accordingly, the authors suggested that the superoxide radical-scavenging effect of the royal jelly might be attributed to antioxidative compounds different from SOD.

Guo et al. [112] found strong antioxidant properties of peptides obtained after the hydrolysis of royal jelly proteins using protease N. The antioxidative properties of the obtained peptides were examined in terms of mechanisms such as hydrogen peroxide, superoxide, and hydroxyl radical-scavenging activities and metal-chelating activity. Twelve obtained peptides showed strong hydroxyl radical-scavenging activity, and three dipeptides containing Tyr residues at their C-termini (Lys-Tyr, Arg-Tyr, and Tyr-Tyr) had strong hydrogen peroxide-scavenging activity. However, in this study, no significant metal-chelating and superoxide anion-radical-scavenging activities of the isolated peptides were noted. The authors concluded that di- and tri-peptides could possess greater antioxidative activity than their constituent amino acids.

### 4.3. Antioxidant Effect of Royal Jelly in Human and Animal Diabetes Mellitus Model

Despite antioxidant properties of the royal jelly found in both *in vitro* and *in vivo* models, there are only a few human studies confirming its effectiveness. The research recently conducted concerned its influence on the parameters associated with diabetes and oxidative stress in people with diabetes mellitus type 2 [114, 115]. In the study conducted by Pourmoradian et al. [114], 50 female volunteers with type 2 diabetes were randomly supplemented with RJ (1000 mg once a day) or placebo for 8 weeks. Before and after the intervention, glycemic and antioxidative-oxidative blood parameters were determined. After the supplementation decreased fasting blood glucose (FBG) and serum glycosylated hemoglobin (HbA1c) levels as well as increased insulin concentration were noticed in the royal jelly-supplemented group in comparison with the placebo one. Moreover, the supplementation caused a significant increase in erythrocyte SOD and GPx activities as well as a decrease in MDA concentration. Similar results were reported by Shidfar et al. [115]. In their study, 46 type 2 diabetic patients were randomly assigned to royal jelly (1000 mg, 3 times a day, for 8 weeks) or placebo -supplemented groups. In the supplemented group, decreased homeostasis model assessment for insulin resistance (HOMA-IR) and increased total antioxidant capacity in comparison with the placebo group were noted. Also in studies using an animal model of diabetes, the improvement of oxidative-antioxidant (MDA, CAT, and ferric-reducing properties of plasma (FRAP)) and biochemical parameters (alanine aminotransferase (ALT), aspartate aminotransferase (AST), alkaline phosphatase (ALP), and fasting blood glucose (FBG)) as well as histopathological changes (tubular differentiation index, mononuclear immune cells, tunica albuginea thickness, seminiferous tubules diameter, Johnsen's score, spermiogenesis index, Sertoli cell index, and meiotic index) were observed after royal jelly supplementation [116, 117]. The authors suggested that their results confirmed the role of reactive oxygen species, even if only secondary, in the pathogenesis of type 2 diabetes. According to them, the royal jelly can ameliorate insulin resistance via antioxidant effect. Based on their results, the authors stated that supplementation with the royal jelly might be beneficial for diabetic patients, but further studies are necessary to clarify the exact mechanism of RJ influence on diabetic parameters.

### 4.4. Antioxidant and Neuroprotective Effects of Royal Jelly

There are several studies that focused on the relationship between the antioxidant and neuroprotective effects of royal jelly, in the literature data. Mohamed et al. [118] investigated the possible neurotoxic effect of tartrazine, a commonly used synthetic azo dye, as well as the potential modulatory role of royal jelly. The group of rats receiving only tartrazine showed not only disturbances of antioxidant biomarkers but also numerous apoptotic cells in the brain cortex and significant decrease in the concentration of the brain neurotransmitters (GABA, dopamine, and serotonin). The authors revealed that the cotreatment of rats with royal jelly improved antioxidant biomarkers as well as neurotransmitter levels. Interestingly, royal jelly also had an activating effect on the central nervous system represented by the reduced degree of damage and apoptosis of brain tissue. The authors concluded that a component responsible for these changes could be 10-hydroxy-2-decenoic acid, because it was demonstrated that in addition to its antioxidative properties, 10H2DA could support the generation of neurons.

The relationship between the neutralizing effect of royal jelly on oxidative stress and neurotoxicity was also sought by Aslan et al. [119]. The researchers revealed that royal jelly diminished the secondary neuronal damage after experimental spinal cord injury in rabbits. In this study, the authors noticed that the treatment with royal jelly prevented lipid peroxidation and augmented endogenous enzymic or nonenzymic antioxidative defense systems levels (Table 4). Moreover, royal jelly treatment significantly decreased the apoptotic cell number induced by spinal cord injury. Because the authors noted statistically higher levels of ascorbic acid in the royal jelly group (laminectomy +100 mg/kg RJ *p.o.*) in comparison with the control group (laminectomy + single dose of 1 mL/kg saline *p.o.*), they suggested that the protective effect of royal jelly against oxidative stress might be related to restoration of ascorbic acid availability.

A study conducted by Teixeira et al. [120] also suggested the existence of antioxidant and neuroprotective effects of royal jelly but in the resistant and cold stress condition (Table 4). The authors postulated that the antioxidant activity of royal jelly, observed in the region of striatum, might correspond to adenosine monophosphate (AMP) N1-oxide—the unique compound of RJ—which can regulate neuronal functions through receptors predominantly expressed in striatum (A2A adenosine receptors). They speculated that the activation of these receptors can prevent radical formation and apoptosis.

### 4.5. Alleviating Effect of Royal Jelly on Oxidative Stress

Studies evaluating the alleviating effect of royal jelly on oxidative stress were performed using several *in vivo* models. The antioxidant effect of royal jelly in cisplatin-induced spermiotoxicity and nephrotoxicity in rats was investigated by Silici et al. [100, 121]. The researchers attributed antioxidant properties of royal jelly to the presence of substances such as 10-hydroxy-2-decenoic acid and free amino acids including proline (which is suggested to act as an antioxidant due to hydroxyl radical-scavenging activity) as well as cystine and cysteine (participating in the synthesis of effective cellular antioxidant—glutathione). The influence of royal jelly on adverse effects generated by the administration of sodium fluoride at high doses in mice was assessed by Kanbur et al. [111]. The authors explained that the antioxidant royal jelly effect could be associated not only with radical-scavenging effect but also with another indirect effect based on the inhibition of enzymes that catalyze the peroxidation of endogenous lipids as well as the gene expression of cytochrome P450, which is one of the intracellular source of H_2_O_2_, O_2_·^−^ and HO· radicals [122]. Other studies also demonstrate the antioxidant effect of royal jelly in an animal model under oxidative stress condition induced by substances like carbon tetrachloride (industrial solvent) [123], azathioprine (immunosuppressive drug) [124], bleomycin [125], methotrexate [126], paclitaxel [127], taxol [128] (chemotherapeutic agents), and oxymetholone (synthetic androgen analogue) [129]. In all mentioned studies, the antioxidant effect of royal jelly consisted of a positive effect on the oxidative-antioxidative parameters (Table 4). In addition, some researchers reported other therapeutic effects of royal jelly such as hepatoprotective [123, 124], cardioprotective [127], or anti-inflammatory [113, 130] ones. The postulated hypotheses explaining the antioxidant effect are the restoration of ascorbic acid availability by royal jelly, regulation of retinol loss [123], antioxidant effect of some free amino acids [124], or radical-scavenging activities of RJ and its component [129].

The antioxidant effect of royal jelly has also been confirmed by *in vitro* studies. For instance, the aim of the study by Inoue et al. [131] was to investigate protective effects of royal jelly fatty acid derivative (4-hydroperoxy-2-decenoic acid ethyl ester (HPO-DAEE)) on oxidative stress-induced cell death using human neuroblastoma SH-SY5Y cells (Table 4). The researchers noted that the pretreatment with HPO-DAEE protected against 6-hydroxydopamine- (6OHDA-) induced cell death by increasing the expression of antioxidant enzyme—heme oxygenase-1 (HO-1) mRNA—through Nrf2-ARE signaling. Interestingly, the authors revealed that the treatment with HPO-DAEE rapidly induced reactive oxygen species generation in SHSY5Y cells. In conclusion, these results suggested that sublethal oxidative stress caused by HPO-DAEE is essential for the activation of this pathway, which is aimed at antioxidant defense. Moreover, the authors observed that HPO-DAEE promoted the phosphorylation of eukaryotic initiation factor 2a (eIF2a), and the subsequent nuclear accumulation of the activating transcription factor-4 (ATF4). The ATF4 pathway is known to be activated under several stress conditions. It is supposed that the interaction of the Nrf2-ARE pathway with the eIF2a-ATF4 pathway augments HO-1 expression.

The investigation of protective effect of royal jelly in the redox state of ovine oocytes matured *in vitro*, and embryonic development following *in vitro* fertilization was performed by Eshtiyaghi et al. [132]. The authors explained that the improvement of oocyte maturation in the case of cells supplemented with royal jelly might be associated with the improvement of redox status. One of the objectives of this study was to examine the effect of different concentrations of royal jelly (2.5, 5, and 10 mg/mL of maturation media) on the *in vitro* maturation and glutathione (GSH) level of ovine oocyte as well as the abundance mRNA of antioxidant enzymes in both oocyte and cumulus cells. Moreover, the authors investigated glucose metabolism-related genes in cumulus cells. The parameters were evaluated following 24 hours of *in vitro* maturation. The authors noted that the dose of 10 mg/mL of RJ not only led to an increase in the number of oocytes but also caused an increase in the intracellular GSH content compared to the control group and the group receiving the lowest dose of royal jelly. In addition, supplementation with 10 mg/mL of royal jelly increased the mRNA GPx in both oocyte and cumulus cells as well as SOD expression in the cumulus cells. However, royal jelly supplementation did not influence mRNA CAT level in both oocyte and cumulus cells. Moreover, the increased expression of phosphofructokinase and glucose 6-phosphate dehydrogenase in the cumulus cells after the addition of royal jelly to the maturation media indicated that the observed protective effect of royal jelly might be related to the activation of glucose metabolic pathways in the surrounding cumulus cells.

The negation of the above results seems to be a study carried out by Filipič et al. [133]. The purpose of this study was to investigate the influence of royal jelly and its bioactive component—10H2DA—and human interferon-alpha (HuIFN-*α*N3—a protein with antiviral, antiproliferative, and antitumor activities—on the proliferation of human colorectal adenocarcinoma cells (CaCo-2) and the oxidative stress parameters—GSH and MDA concentration. Royal jelly and HuIFN-*α*N3 applied at 2 : 1 ratio and royal jelly applied in combination with 10H2DA (2 : 1 ratio) caused a decrease in the level of GSH and increase in lipid peroxidation indicator level (MDA) in CaCo-2 cells in comparison with the control group. On the other hand, it was observed that these combinations had the highest antiproliferative effect. The authors suggested that antiproliferative effects of RJ, HuIFN-*α*N3, and 10H2DA on the CaCo-2 cells could be connected not only with the induction of apoptosis and cytotoxicity but also with their influence on the prooxidative-antioxidative balance.

Attempts to explain the alleviating mechanism of royal jelly's action on nitrosative stress were made by Sugiyama et al. [134]. Researchers examined the ability of 10H2DA to inhibit LPS-induced nitric oxide (NO) generation using the RAW264 murine macrophage cell line. Their study was based on the fact that LPS stimulates the production of interferon- (IFN-) *β*, induction of IFN regulatory factor-1, and activation of IFN-stimulated response element. These factors are required for iNOS (nitric oxide synthases) induction. The authors noted that 10H2DA not only inhibited LPS-induced nitric oxide (NO) generation but also restrained IFN-*β*-induced nuclear factor- (NF-) *κ*B activation and tumor necrosis factor- (TNF-) *α* production. The authors concluded that 10H2DA inhibited LPS- and IFN-*β*-induced NO productions via the inhibition of NF-*κ*B activation induced by LPS or IFN-*β*. Similar studies have been carried out by Takahashi et al. [135]. Using the same cell line, authors noted the inhibition of interferon-*γ*-induced NO production by 10H2DA through the inhibition of interferon regulatory factor-8 induction (Table 4).

## 5. Comparison of the Antioxidant-Related Potential of Propolis, Bee Pollen, and Royal Jelly

Numerous *in vitro* studies using DPPH, ABTS^+^, FRAP, ORAC methods, and so on have confirmed the antioxidant potential of bee products [3, 19, 20, 31, 35, 36, 38, 79, 81, 85, 110]. In this part, we will try to compare the antioxidant capacity of propolis, bee pollen, and royal jelly among each other.

It is well known that the antioxidant capacity of bee products is strongly dependent on their chemical composition. In general, the antioxidant activity of poplar propolis is believed to be largely influenced by both total polyphenol and total flavonoid contents, while the Brazilian one by phenolic compounds but different ones than flavonoids [3, 34–36, 38]. In the case of bee pollen, research has shown divergent results; some have shown a strong positive correlation between the total content of phenolic compounds and antioxidant capacity [79, 81], whereas others have found no considerable relationships [71, 87, 136]. Leja et al. [87] suggested, in turn, that not phenolic compounds but bee pollen phenylpropanoids are responsible for the inhibition of linoleic acid peroxidation. As for royal jelly, its antioxidant properties are mainly attributed to the presence 10-hydroxydecanoic acid and free amino acids including proline as well as cystine and cysteine following phenolic compounds [100, 102, 121].

Basing on literature data, propolis seems to be the most powerful antioxidant among all the analyzed bee products. The comparison of the phenolic compounds' content seems to confirm the above thesis—the highest amount of both total phenols and flavonoids has been found in propolis followed, in order, by bee pollen and royal jelly. However, it should be emphasized that royal jelly also contains other compounds possessing antioxidant character. The next confirmation of the above thesis are the results obtained by Nakajima et al. [122]. In their study, the rank order of antioxidant potencies measured by the hydrogen peroxide, superoxide anion, and hydroxyl radical-scavenging capacities was as follows: water propolis extract, ethanol propolis extract, and ethanol pollen extract, but neither royal jelly nor 10-hydroxy-2-decenoic acid (10-HDA) had any effects. So both propolis extracts showed much greater antioxidant activity than the bee pollen one, and surprisingly, the water propolis extract was much more effective that the ethanol one.

On the other hand, in the *in vivo* study, waterborne bee products (including royal jelly, bee pollen, and propolis) were shown to be able to reverse the oxidative damage caused by exposure to tebuconazole. They affected the brain, kidney, and liver lipid peroxidation, protein carbonylation, and antioxidant markers. All studied products were effective, but the effects were dependent on doses and organs, and no clear trend was observed [12]. Similar results were observed by Turkish researchers [137] who noticed the highest antioxidant capacity as well as total phenolic and flavonoid content in propolis, followed, in order, by pollen, honey, and royal jelly. Despite very significant differences in both phenolic content and antioxidant activities measured using FRAP and DPPH methods, in an *in vivo* study, all products revealed similar hepatoprotective activity against CCl4-induced hepatic damage in rats. All of them had the very similar effect on liver parameters and antioxidant/oxidant markers with only a very slight advantage in the case of propolis. The authors suggested that these results could be explained through their bioavailability to the treated animals. It should be underlined that in the case of bee pollen, research has shown not only its antioxidative but also its prooxidative action. For example, in LPS-stimulated macrophages, the bee pollen extract was shown to efficiently scavenge nitric oxide, although against superoxide, it behaved as antioxidant at lower concentrations and as prooxidant at higher concentrations [138]. To the best of our knowledge, prooxidative properties of propolis and royal jelly have not been found. On the other hand, some studies have found that bee pollen along with propolis exhibits strong antioxidant effects, while royal jelly had no effect.

As for the mechanism underlying the potential antioxidant-related effects, the most studied is propolis. It has been shown the treatment with propolis and its common compound pinocembrin generates an increase in at least one of the enzymatic antioxidant pathway, namely, it induces the translocation of nuclear factor erythroid 2-related factor 2 (Nrf2) to the nucleus and subsequent expression of antioxidant response element- (ARE-) mediated antioxidant genes such has HO-1 and *γ*-GCS [6, 62]. Moreover, propolis or pinocembrin was found to regulate the expression, on both the mRNA and protein levels, of genes encoding other antioxidant markers, including LOX-1, *γ*-GCS, GCLM, GCLC, and TrxR1 [6, 49, 56, 63, 66]. The royal jelly was noticed to affect HO-1 expression through Nrf2-ARE pathway as well as the expression of cytochrome P450, GPx, and SOD [122, 131, 132]. As for bee pollen, to the best of our knowledge, there is no information in the literature data on its influence on the expression of antioxidant-related genes. Furthermore, the mechanisms of bee products for the reversal of oxidative damage appear to involve the reduction of lipid peroxidation (MDA and TBARS) and oxidant parameters (e.g., ROS) as well as the augmentation of antioxidant enzyme activities (e.g., CAT, SOD, GPx, and GST). But this assumption is based on the fact that the treatment with bee products affects the level of the above parameters in blood and studied organs.

Propolis is also the most studied in cell and animal research. It is credited with having antioxidant–related neuroprotective and cardioprotective actions and is thus suggested as a protective agent against Alzheimer's [47, 48] and Parkinson's diseases [6] as well as atherosclerosis [55]. The neuroprotective effect of royal jelly was also noted. All analyzed bee products have been tested in relation to the reduction of the negative effect of chemotherapy—in each case, the obtained results were promising [4, 5, 51, 90, 94, 121, 125, 127, 128]. The rest studies are focused on estimating the protective action of bee products against various harmful factors and drugs causing oxidative stress.

Although *in vitro* and animal studies seem to confirm the antioxidant-related protective effect of bee products, there are only a few studies performed on humans in the literature data [44–46, 114, 115]. The existing ones aim at evaluating the supplementation effect in healthy population or type 2 diabetic patients and involve propolis as well royal jelly. Herein, the obtained results are inconsistent. For example, propolis supplementation had a positive effect in men but not in women [45]. As for diabetic patients, royal jelly seems to have a higher potential than propolis as it was shown to regulate the parameter associated with diabetes HOMA-IR [115], while propolis affected only oxidative-related parameters [46].

Undoubtedly, there is a gap in the literature data considering the evaluation of the bee products' potential in human population. This results from the fact that bee products are characterized by very complicated biological matrix. Moreover, their composition varies depending on many factors, which are sometimes very difficult to control, that is, temperature. In fact, the chemical composition of each sample should be tested before being included in an *in vivo* study. This is very tedious, time-consuming, and troublesome. Therefore, researchers today are more likely to use the commercially available equivalents, for example, CAPFE or pinocembrin. This is not exactly a good direction since we should remember that the complex products may exert synergistic effects. For example, Almaraz-Abarca et al. [93] evaluated the potential of bee pollen and chosen phenolic compounds in the inhibition of lipid peroxidation in microsomal preparations of mouse liver and showed much higher effectiveness of bee pollen (about 5.5 times as high as quercetin alone, 3 times as its glucoside (quercitrin) alone, and 2.4 times as caffeic acid alone).

## 6. Conclusion

The aforementioned *in vitro* and animal studies seem to confirm the usefulness of using bee products (propolis, bee pollen, and royal jelly) as natural agents capable of counteracting the effects of oxidative stress underlying the pathogenesis of numerous diseases or disorders, such as neurodegenerative disorders, cancer, diabetes, and atherosclerosis, as well as negative effects of different harmful factors and drugs (e.g., cytostatic agents). However, studies on their role in humans are very limited, and the existing ones have aimed mostly at evaluating the effect of the supplementation of commercially available extracts of propolis or royal jelly in healthy people or type 2 diabetes. Unfortunately, in the available literature, there is a lack of studies considering this issue in the context of neurodegenerative disorders or cancers, although promising results were obtained in animal studies. This may result from the fact that particular samples of bee products may have different compositions, so it is difficult to draw a general conclusion concerning their potential therapeutic application without a detailed chemical analysis.

In conclusion, future studies concerning the question if bee products could be a promising adjuvant in the therapy of oxidative stress-related disorders or diseases in human seem to be advisable.

## Figures and Tables

**Table 1:** The results of the research on the dependence between the solvent used for the extraction and the antioxidant properties of the obtained propolis and bee pollen extracts.

Source	Type of bee products	The used solvents	The dependence between the used extraction solvent and the properties of the obtained extract
Bittencourt et al. [31]	Green and brown Brazilian propolis	Ethanol (95%) extraction, evaporation, and dissolving in 80% ethanol and then partitioning with hexane or dichloromethane.	Antioxidant activity showed considerable differences depending on the used solvent and propolis type. In DPPH assay, the strongest antioxidant activity was found in dichloromethane and ethanol extracts of green propolis as well as dichloromethane extract of brown propolis with the IC50 values at least two times less than in the other cases, namely, in hexane extract of brown propolis, ethanol extract of brown propolis, and hexane extract of green propolis.
Narimane et al. [34]	Algerian propolis collected from Beni Belaid, Jijel (northeast of Algeria)	CH_2_Cl_2_-MeOH (1 : 1, *v*/*v*) extraction followed by MeOH-H_2_O (70 : 30, *v*/*v*) one, concentrating and dissolving in boiling water. Then the water solution underwent successive extraction by using of chloroform, ethyl acetate, and *n*-butanol.	The DPPH, ABTS, FRAP, and CUPRAC methods were applied to determine antioxidant activity. The ethyl acetate and n-butanol extracts proved to be the most effective ones.
Sun et al. [3]	Beijing propolis	Water, 25%, 50%, 75%, 95%, and 100% ethanol.	The 75% extract demonstrated the highest antioxidant capacity measured by DPPH, ABTS, FRAP, ORAC, and CAA methods.
LeBlanc et al. [11]	Six pollen types (mesquite, yucca, palm, terpentine bush, mimosa, and chenopod) collected in Arizona between March and November	Water, methanol, ethanol, propanol, 2-propanol, acetone, dimethylformamide, and acetonitrile.	Antioxidant activity showed considerable differences depending on the used solvent. In FRAP assay, methanol and dimethylformamide extracts displayed the greatest activity values, whereas those obtained with the application of acetonitrile displayed the lowest ones. Acetone extracts were also found to possess low activity in the case of most types of bee pollen. Similar results were obtained with using DPPH assay.
Silva et al. [41]	Pollen loads collected from *Melipona rufiventris* (stingless bees) colonies in Brazil	Fractional extraction with subsequent using of ethanol, n-hexane, and ethyl acetate.	The DPPH method was applied to determine antioxidant activity. The ethyl acetate extract proved to be the most effective one. The ethanol extract showed much less (more than six times) efficiency, whereas the capacity of the hexane extract was slight (practically inactive).
Chantarudee et al. [42]	Bee pollen collected in Thailand in the summer (June), its main component being identified as pollen of corn (*Z. mays* L.)	Subsequent application of 80% methanol, dichloromethane, and hexane.	The antioxidant activity of the obtained extracts was estimated by DPPH assay. The hexane extract proved to be completely inactive, whereas that obtained with using dichloromethane showed the best antioxidant properties, comparable even with the reference antioxidant—ascorbic acid.
Maruyama et al. [43]	Bee pollen from *Cistus* sp. of Spanish origin. Bee pollen from *Brassica* sp. of Chinese origin	Water and 95% ethanol	In rats with induced hind paw edema, the oral administration of bee pollen water extract had practically no effect, while ethanol extract displayed the greatest effectiveness in the inhibition of paw edema.

**Table 2:** The protective effects of propolis against prooxidant action of different harmful factors.

Source	Toxic or harmful factor	Harmful effects of an applied factor	The type of propolis and the way of application	Effects of propolis coadministration
Neuroprotective effect of propolis				
Bazmandegan et al. [9]	Cerebral ischemia-induced oxidative injury in a mouse model of stroke	↑ MDA ↑ SOD and SOD/GPx ratio ↓ GPx in brain	Water-extracted Iran brown propolis; from two regions of Iran; 100 and 200 mg/kg; *i.p.* at 48, 24, and 1 h before and 4 h after the induction of ischemia	↓ MDA ↓ SOD and SOD/GPx ratio ↑ GPx^∗∗^ in brain
Ni et al. [47]	H_2_O_2_-induced neurotoxicity, human neuroblastoma SH-SY5Y cells (100 *μ*M for 4 h or 1 h for ROS)	↑ ROS in mitochondria ↑ 8-oxo-dG, the DNA oxidative damage marker ↓ Cell viability	Methanol extract of Brazilian green propolis, 10 *μ*g/mL pretreatment for 2 h (or 1 h for ROS)	↓ ROS in mitochondria ↓ 8-oxo-dG ↑ Cell viability
Nanaware et al. [48]	*β*-Amyloid 25–35-induced Alzheimer's disease model in rats, (10 *μ*g/rat injected bilaterally)	↓ SOD, GSH, CAT, NO ↑ MDA in brain	Macerated ethanolic extract of Indian propolis; 100, 200, and 300 mg/kg b.w., *p.o*. (posttreatment after 14 days); 21 days	↑ SOD, GSH, CAT, NO ↓ MDA in brain All doses were effective; the effect slightly increased with increasing dose
Jin et al. [6]	6-Hydroxydopamine-induced oxidative stress in human neuroblastoma SH-SY5Y cells (50 *μ*M for 24 h)	↑ ROS ↑ MDA ↓ SOD ↓ Bcl-2/Bax ratio	Pinocembrin; 1, 5, and 25 *μ*M pretreatment for 4 hours	↓ ROS^∗∗^ ↓ MDA^∗∗^ ↑ SOD^∗∗^ ↑ Bcl-2/Bax ratio^∗∗^ ↑ Nrf2 translocation^∗∗^ ↑ HO-1 and *γ*-GCS expression^∗∗^
de Oliveira et al. [49]	Paraquat-induced neurotoxicity in SH-SY5Y cells (100 *μ*M, 24 hours)	↑ O_2_^−•^ production, lipid peroxidation, protein carbonylation, and protein nitration in mitochondrial membranes ↓ Thiol content in mitochondrial membranes ↓ GSH in mitochondria	Pinocembrin; 25 *μ*M pretreatment for 4 hours	↓ O_2_^−•^ production, lipid peroxidation, protein carbonylation, protein nitration, as well as oxidation of thiol groups in mitochondrial membranes ↑ Thiol content in mitochondrial membranes ↑ GSH in mitochondrial membranes ↑ Erk1/2-Nrf2 axis ↑ GCLM, GCLC, GSH, and HO-1
Barros Silva et al. [7]	6-OHD-induced dopaminergic neuronal loss in rats, (3 *μ*L, 8 mg/mL, *s.i*.)	↑ Hydrogen peroxide in striatum ↑ Cu, Fe, Mn, and Zn in brain	Caffeic acid phenethyl ester (CAPE); 10 *μ*M/kg, *i.p*., cotreatment for 5 days	↓ Hydrogen peroxide in striatum ↓ Cu, Fe, Mn, and Zn in brain
Mahmoud et al. [50]	K_2_CrO4-induced neurotoxicity in rats, (2 mg/kg b.w. for 30 days, *i.p*.)	↑ MDA and NO ↓ SOD, GPx, and GSH in cerebrum ↑ JAK2, STAT3, and SOCS3 mRNA and protein in cerebrum	CAPE 20 mg/kg b.w. cotreatment for 30 days, orally	↓ MDA and NO ↑ SOD, GPx, and GSH in cerebrum ↓ JAK2, STAT3, and SOCS3 mRNA and protein in cerebrum
Propolis role in mitigation of chemotherapy side effect				
Kumari et al. [51]	Mitomycin C-induced testicular toxicity in male mice, (8 mg/kg b.w., *i.p*., single dose)	↑ MDA ↓ GSH, SOD, and CAT in testicular cells	Hydroethanolic extract of Indian propolis pretreatment (1 h prior) 400 mg/kg, *i.p*., single dose	↓ MDA ↑ GSH and CAT in testicular cells
Alyane et al. [5]	Doxorubicin-induced toxicity in rat heat mitochondria, (20 mg/kg b.w., *i.p*., single dose)	↑ Mitochondrial MDA ↓ RCR (respiratory chain ratio) and P/O ratio ↑ O_2_^−^ (evaluated *in vitro*)	Propolis extract pretreatment with 100 mg/kg/day, *p.o.* for four days prior	↓ Mitochondrial MDA ↑ RCR (respiratory chain ratio) and P/O ratio ↓ O_2_^−^ (evaluated *in vitro*)
Propolis as a modulator of cardiovascular disease markers				
Salmas et al. [52]	N*ω*-nitro-L-arginine methyl ester- (L-NAME-) induced hypertension in rats, (40 mg/kg b.w.; *i.p.* for 28 days)	↓ TAS, PON1 ↑ TOS, ADMA, and NF-*κ*B	Propolis CAPE coadministration: propolis: 200 mg/kg/d; 28 days, by gavage; CAPE: 50 *μ*M/kg/d; 14 days, *i.p.*	↑ TAS ↑ PON1—only propolis ↓ TOS, ADMA ↓ NF-*κ*B—only propolis
Ahmed et al. [53]	Isoproterenol-induced myocardial infarction in rats, (85 mg/kg injection for 2 days—on the 29th and 30th days)	↓ SOD, GPx, GRx, and GST in myocardium ↑ TBARS in myocardium	Malaysian propolis ethanol extract, pretreatment with 100 mg/kg/day, orally, 30 days	↑ GPx, GRx, and GST in myocardium ↓ TBARS in myocardium
Sun et al. [54]	H_2_O_2_-induced rat cardiomyocytes (H9c2) oxidative injury, (700 *μ*M, 6 h)	↑ MDA ↓ SOD and GPx	CAPE, benzyl caffeate, and cinnamyl caffeate pretreatment with 1, 5, and 10 *μ*M for 12 h	↓ MDA—doses of 5 and 10 *μ*M ↑ SOD and GPx—doses of 5 and 10 *μ*M
El-Awady et al. [58]	High glucose-induced vascular endothelial dysfunction, isolated rat aorta, (44 mM for 3 hours)	↑ TBARS in rat aorta ↓ SOD and GSH in rat aorta	Propolis extract pretreatment 400 *μ*g/mL, 30 min prior	↓ TBARS in rat aorta ↑ SOD and GSH in rat aorta
Propolis as protective agent against prooxidants' toxicity				
Yonar et al. [59]	Trichlorfon-induced oxidative stress in fish, environmental exposure, 11 and 22 mg/L, 14 days	↑ MDA in the liver, kidney, and gill ↓ GSH, SOD, CAT, and GPx in the liver, kidney and gill	Propolis cotreatment 10 mg/kg of fish weight, 14 days	↓ MDA in the liver, kidney, and gill ↑ GSH, SOD, CAT, and GPx in the liver, kidney, and gill
Ferreira et al. [12]	Tebuconazole-induced oxidative stress in fish, environmental exposure (0.88 mg/L)	↑ MDA and carbonyl protein in brain, liver, and kidney ↑ GST in liver ↓ GST in brain ↑ CAT in kidney and brain ↓ SOD in liver	Propolis; 0.01, 0.05, and 0.1 g/L	↓ MDA and carbonyl protein in brain, liver and kidney ↑ GST in brain, liver^∗∗^ and kidney ↑ CAT in liver^∗∗^ ↓ CAT in kidney^∗∗^ and brain ↑ SOD in liver
Aksu et al. [60]	Paracetamol- (PRC-) induced reproductive toxicity in rats, (500 mg/kg b.w., by oral gavage)	↓ SOD, CAT, GPx, and GSH in testicular tissue ↑ MDA in testicular tissue	Chrysin; pretreatment with 25 mg/kg and 50 mg/kg b.w., by oral gavage, 7 days	↑ GSH, CAT^∗∗^, GPx^∗∗^, SOD (only the higher dose) in testicular tissue ↓ MDA in testicular tissue
Manzolii et al. [61]	Methylmercury-induced oxidative stress (30 *μ*g/kg b.w., by gavage, 45 days)	↓ GSH in blood	Chrysin; cotreatment (0.10, 1.0, and 10 mg/kg b.w., by gavage, 45 days)	↑ GSH in blood
Saito et al. [62]	UVA irradiation, human skin fibroblast cells—NB1-RGB (10 J/cm^2^)	↑ HO-1 expression	Brazilian green propolis; 3, 10, or 30 *μ*g/mL 3,5-di-*O*-caffeoylquinic acid, 3,4-di-*O*-caffeoylquinic acid, and chlorogenic acid; 1 or 3 *μ*g/mL	↑ HO-1 expression ↑ Nrf2 nuclear translocation to the nuclei (only propolis extract was studied)
Cao et al. [63]	H_2_O_2_-induced oxidative stress, mouse L929 fibroblast cell lines, (600 *μ*M H_2_O, 12 hours)	↑ ROS ↓ Cell viability	Ethanol extract of Chinese propolis; pretreatment with 5, 7.5, and 10 *μ*g/mL per 3 hours prior	↓ ROS^∗∗^ ↑ Cell viability^∗∗^ ↑ HO-1, GCLM, and GCLC at mRNA level (the highest dose was studied) ↑ HO-1 and GCLM at protein level (the highest dose was studied)
Arabameri et al. [64]	Maternal separation-induced stress, the neonatal rats, separated 6 hours per day, 15 days	↑ MDA in ovarian tissue ↓ SOD, GPx, and FRAP in ovarian tissue	Iranian propolis; cotreatment 50, 100, or 200 mg/kg b.w.; 15 days	↓ MDA^∗∗^ in ovarian tissue ↑ SOD^∗∗^, GPx, and FRAP^∗∗^ in ovarian tissue All three doses exerted a positive effect, but the most effective was 200 mg/kg
Zhang et al. [66]	H_2_O_2_-induced oxidative stress, RAW264.7 cells, 300 *μ*M for 13 hours	↑ Intracellular ROS	Two ethanol extracts of Chinese propolis, pretreatment for 0.5 hour before	↓ Intracellular ROS
	RAW264.7 cells not subjected to any factor	————	Two ethanol extracts of Chinese propolis	↓ Intracellular ROS ↑ HO-1, GCLM, and TrxR1 on both the mRNA^∗∗^ and protein levels^∗∗^ The most effective for HO-1

ADMA: asymmetric dimethylarginine; Bax: Bcl-2-related ovarian killer protein; Bcl-2: B-cell lymphoma 2; CAT: catalase; GCLC: glutamate-cysteine ligase catalytic subunit; GCLM: glutamate-cysteine ligase regulatory subunit; Erk 1/2: extracellular signal-regulated kinase ½, FRAP: ferric reducing ability; GPx: glutathione peroxidase; GRx: glutathione reductase; GSH: reduced glutathione; GST: glutathione reductase; HO-1: heme oxygenase-1; JAK 2: Janus kinase 2; MDA: malondialdehyde; NF-*κ*B: nuclear factor kappa B; Nrf2: nuclear factor erythroid 2-related factor 2 (Nrf2); NO: nitric oxide; 8-oxo-2′-deoxyguanosine, P/O: phosphate/oxygen ratio; PON1: paraoxonase; RCR: respiratory control ratio; ROS: reactive oxygen species; STAT3: signal transducer and activator of transcription 3; SOCS3: suppressor of cytokine signaling 3; SOD: superoxide dismutase; TAS: total antioxidant status; TBARS: thiobarbituric acid reactive substances; TOS: total oxidant status; TrxR1: thioredoxin reductase 1; *γ-GCS: γ*-glutamylcysteine synthetase. ↓: decrease; ↑: increase; ^∗∗^the effect depended on used dose.

**Table 3:** The protective effects of bee pollen against prooxidant action of different harmful factors.

Source	Toxic factor	Harmful effects of a toxic factor	The type of bee pollen and the way of application	Effects of bee pollen coadministration
Mitigation effect of bee pollen on chemiotherapeutic agents				
Huang et al. [88]	Cisplatin-induced toxicity in rats, (8 mg/kg b.w. *i.p.* in single dose) on the 7th day of the 12-day-experiment	↑ MDA and iNOS: liver and kidney ↓ SOD, CAT, and GSH: liver and kidney	*Schisandra chinensis* bee pollen extracted with 70% ethanol, 400, 800, and 1200 mg/kg b.w. *p.o.,* 12 days	↓ MDA in liver^∗∗^ and kidney ↓ iNOS in liver and kidney^∗∗^ ↑ SOD in liver^∗∗^ and kidney^∗∗^ ↑ CAT in the liver and kidney ↑ GSH in the liver and kidney
Tohamy et al. [89]	Cisplatin-induced toxicity in male mice (2.8 mg/kg b.w. *i.p*. twice/week for 3 weeks)	↑ Lipid peroxidation in liver, kidney and testis ↓ CAT and GSH in the liver, kidney, and testis	Water, Egyptian bee pollen extract, 140 mg/kg b.w. once a day orally, during the last 2 weeks of cisplatin exposure	↓ Lipid peroxidation in the kidney, liver, and testis ↑ CAT and GSH in the kidney, liver, and testis
Mitigation effect of bee pollen on other toxic agents				
Ferreira et al. [12]	Tebuconazole-exposed fish (catfish jundiá), 0.88 mg/L (16.6% of 96 h LC_50_) 96 hours	↑ Lipid peroxidation in the liver, kidney, and brain ↓ SOD in liver ↑ CAT in the liver and brain	Bee pollen; 0.01, 0.03, and 0.05 g/L, environmental exposure	↓ Lipid peroxidation in the liver, kidney^∗∗^, and brain^∗∗^ ↑ SOD in the liver ^∗∗^ ↓ CAT in liver^∗∗^: low and high doses ↓ CAT in brain^∗∗^
Yıldız et al. [1]	Carbon tetrachloride-induced hepatotoxicity in rats (0.85 mL/kg b. w. *i.p*., 7 days)	↑ plasma ALT and AST ↑ MDA in liver, RBC and plasma; ↓ SOD in plasma, RBC and liver	Bee pollen collected during flowering season in Turkey (Western Black Sea region) with dominant component chestnut *sativa* pollen (>45%), 200 mg/kg/day orally, 400 mg/kg/day orally, 7 days	↓ Plasma ALT: high dose ↓ Plasma AST ↓ MDA in the plasma, RBC, and liver
Almaraz-Abarca et al. [93]	Bromobenzene-induced hepatotoxicity in mice, 94.211 *μ*g/mL in oil, 200 *μ*L orally	↑ Lipid peroxidation liver	Bee pollen from mesquite (*Prosopis juliflora*) collected in April in Mexico, extracts of two flavonol concentration (9.794 *μ*g/mL and 21.751 *μ*g/mL), 200 *μ*L orally	↓ Liver lipid peroxidation—only the higher dose
Ketkar [92]	Chronic exercise-induced oxidative stress in rats, 4 weeks	↓ Gastrocnemius muscle SOD and GSH ↑ Gastrocnemius muscle MDA and NO ↓ Weight of gastrocnemius muscle and body	The neat and processed (1 mg of bee pollen : 500 mg of Captex 355 : 750 mg of Tween 80) monofloral Indian mustard bee pollen, 100, 200, or 300 mg/kg daily, orally	↑ SOD and GSH in gastrocnemius muscle ↓ MDA in gastrocnemius muscle: neat^∗∗^: high dose, processed: all doses ↓ NO in gastrocnemius muscle neat^∗∗^: higher ones, processed: all doses ↑ Body weight ↑ Gastrocnemius muscle weight neat^∗∗^: high one, processed^∗∗^: higher doses The positive effects increase along with the increase in the dose

ALT: alanine aminotransferase; AST: aspartate aminotransferase; CAT: catalase; GSH: reduced glutathione, iNOS: inducible nitric oxide synthase; MDA: malondialdehyde; NO: nitrogen oxide; RBC: red blood cell; SOD: superoxide dismutase; ↓: decrease; ↑: increase; ^∗∗^the effect depended on used dose.

**Table 4:** The protective effects of royal jelly against prooxidant action of different harmful factors.

Source	Toxic/harmful factor	Harmful effects of an applied factor	The dose and the way of application of royal jelly or its ingredients	Effects of royal jelly or its ingredient coadministration
Neuroprotective effect of royal jelly				
Mohamed et al. [118]	Tartrazine-induced neurotoxicity in rats (500 mg/kg *p.o.*, 30 days)	↑ MDA; ↓ SOD, CAT, and GSH in brain tissue	RJ: 300 mg/kg *p.o.*, 30 days	↓ MDA; ↑ SOD, CAT, and GSH in brain tissue
Aslan et al. [119]	Neuronal damage after experimental spinal cord injury (laminectomy) in rabbits	↓ Nitrate and nitrite in serum ↓ SOD and GPx; ↑ CAT in erythrocytes ↑ MDA, nitrite, and nitrate, ↓ GSH in cerebrospinal fluid ↑ MDA and GSH in brain tissue	RJ: 100 mg/kg b.w. *p.o.* after trauma	↓ MDA, ↑GSH in whole blood ↑ Nitrate, Vit. C, retinol, and *β*-carotene in serum ↑ SOD, CAT, and GPx in erythrocytes ↓ MDA and nitrite; ↑ GSH in cerebrospinal fluid ↓ MDA; and ↑ GSH in brain tissue
Teixeira et al. [120]	Resistant and cold stress condition	↑ TBARS brain, cerebellum, cerebral cortex, and hippocampus ↓ GPx, GR, G6PDH, and GSH in the brain and striatum	RJ: 200 mg/kg by gavage, 14 days	↓ TBARS level in the brain, cerebellum, striatum, and hippocampus ↑ GPx, GR, G6PDH, and GSH concentration in cerebral cortex and striatum
Inoue et al. [131]	6-Hydroxydopamine- (6OHDA-) induced cell death; human neuroblastoma SH-SY5Y cells	↑ ROS generation	RJ fatty acid derivative—HPO-DAEE: 50 *μ*M	↑ Expression of HO-1 mRNA ↑ Cell viability ↓ ROS generation
Mitigation effect of royal jelly on chemotherapeutic agents				
Silici et al. [100]	Cisplatin-induced spermiotoxicity in rats (7 mg/kg b.w. *i.p.* in single dose)	↑ MDA ↓ SOD, CAT, and GPx in testis tissues	RJ pretreatment and posttreatment: 50 or 100 mg/kg b.w. *p.o.* once a day, for 10 days	↓ MDA ↑ SOD, CAT, and GPx in testis tissues
Silici et al. [121]	Cisplatin-induced nephrotoxicity in rats (7 mg/kg *i.p.* in single dose)	↑ MDA ↓ SOD, CAT, and GPx in renal tissues	RJ pretreatment and posttreatment: 50 or 100 mg/kg b.w. *p.o.* once a day, for 10 days	↓ MDA ↑ SOD, CAT, and GPx in renal tissues
Amirshahi et al. [125]	Bleomycin-induced spermiotoxicity in rats (10 mg/kg b.w., 48 days, twice a week, *i.p.*)	↑ MDA in testicular tissue	RJ: 100 mg/kg b.w., *p.o.*, 48 days	↓ MDA in testicular tissue
Kaynar et al. [126]	Methotrexate-induced oxidative stress in rats (20 mg/kg b.w. *i.p.*, single dose)	↑ MDA and ↓ SOD and GPx in plasma	RJ: 50 or 100 mg/kg b.w., *p.o*, 10 days	↓ MDA and ↑ SOD^∗∗^ and GPx^∗∗^ in plasma
Malekinejad et al. [127]	Paclitrexal-induced cardiotoxicity in rats (7.5 mg/kg b.w. *i.p.*, weekly, 7 weeks)	↓ TAC in serum ↑ MDA and NO in heart tissue	RJ: 50, 100, or 150 mg/kg b.w., *p.o.*, 28 days	↑ TAC^∗∗^ in serum ↓ MDA^∗∗^ and NO in heart tissue
Delkhoshe-Kasmaie et al. [128]	Taxol-induced damage of the testis (7.5 mg/kg b.w. *i.p.*, weekly, 4 weeks)	↑ MDA and NO and ↓ TTM in testis tissue	RJ: 50, 100, or 150 mg/kg b.w., 4 weeks	↓ MDA and NO^∗∗^ and ↑ TTM^∗∗^ in testis tissue
Mitigation effect of royal jelly on other toxic agents				
Kanbur et al. [111].	Sodium fluoride-induced oxidative stress in mice (200 ppm fluoride *p.o.*, 7 days)	↑ MDA in erythrocytes and liver tissue ↓ SOD, CAT, and GPx in erythrocytes ↑ GPx, ↓ CAT, and SOD in the liver tissue	RJ: 50 mg/kg b.w. by gavage for 7 days	↓ MDA in erythrocytes and liver tissue ↑ SOD and CAT in erythrocytes and liver tissue ↓ GPx in erythrocytes
Cemek et al. [123]	Carbon tetrachloride-induced acute liver damage in rats (0.8 mL/kg b.w. *s.c.*, 20 days)	↑ MDA in the whole blood, liver, brain, kidney, lung, and heart tissues ↓ GSH in the whole blood ↓ Vit. C, *β*-carotene, and retinol in serum	RJ: 50, 100, and 200 mg/kg b.w., *p.o.*, 20 days	↓ MDA in the whole blood, liver^∗∗^, brain^∗∗^, kidney, lung, and heart tissues ↓ GSH in the whole blood^∗∗^ ↑ GSH in the liver and brain tissues^∗∗^ ↑ Vit. C, *β*-carotene, and retinol in serum
Ahmed et al. [124]	Azathioprine-induced toxicity in rats (50 mg/kg b.w. *i.p.*, single dose)	↑ MDA and GSH in the liver tissue	RJ: 200 mg/kg *p.o.*, 7 days	↓ MDA and ↑ GSH in the liver tissue after 24 h and 2 weeks of posttreatment
Ghanbari et al. [116]	Streptozotocin-induced diabetes mellitus (60 mg/kg b.w., *i.p.*)	↑ MDA, ↓ CAT, and FRAP in the liver and pancreas	RJ: 200 mg/kg b.w., *p.o.*, 6 weeks	↓ MDA in the liver and pancreas ↑ CAT and FRAP in the liver and pancreas
Ghanbari et al. [117]	Streptozotocin-induced diabetes mellitus (50 mg/kg b.w., *i.p.*)	↓ CAT and FRAP in testicular tissue	RJ: 200 mg/kg b.w., *p.o.*, 6 weeks	↑ CAT and FRAP in the testicular tissue
Sugiyama et al. [134]	LPS- and interferon-*β*-induced NO generation; RAW264 murine macrophage cell line	↑ Nitrate ↑ iNOS promoter activity ↑ NF-*κ*B activation and TNF-*α* production	RJ fatty acid (1 mM, 2 mM, 4 mM 10H2DA)	↓ Nitrate ↓ iNOS promoter activity ↓ NF-*κ*B activation and TNF-*α* production
Takahashi et al. [135]	Interferon-*γ*-induced NO production; RAW264 murine macrophage cell	↑ Nitrate ↑ iNOS promoter activation ↑ NF-*κ*B activation and TNF-*α* production	RJ fatty acid (1 mM, 2 mM, 5 mM 10H2DA)	↓ Nitrate ↓ iNOS promoter activation and NF-*κ*B activation^∗∗^ and TNF-*α* production

10H2DA: 10-hydroxy-trans-2-decenoic acid; CAT: catalase; FRAP: iron reduction capacity; G6PDH: glucose-6-phosphate dehydrogenase; GPx: glutathione peroxidase; GR: glutathione reductase; GSH: reduced glutathione; G6PDH: glucose-6-phosphate dehydrogenase; HPO-DAEE: hydroperoxy-2-decenoic acid ethyl ester; HO-1: heme oxygenase-1; iNOS: inducible nitric oxide synthase; MDA: malondialdehyde; NO: nitric oxide; NF-*κ*B: nuclear factor kappa-light-chain-enhancer of activated B cells; Nrf2/ARE: nuclear factor erythroid 2-related factor 2 (Nrf2)/antioxidant responsive elements (AREs); SOD: superoxide dismutase; TAC: total antioxidant capacity; TBARS: thiobarbituric acid reactive substances; TNF-*α*: tumor necrosis factor alpha; TTM: total thiol molecules; ↓: decrease; ↑: increase; ^∗∗^the effect depended on used dose.
